# Stable Isotopes in Eye Lenses Record Patterns and Variation in Resource‐Use Ontogeny of Three New Zealand Kelp Forest Fishes

**DOI:** 10.1002/ece3.73921

**Published:** 2026-07-09

**Authors:** Joseph S. Curtis, Gretchen J. McCarthy, Leonardo M. Durante, Thomas M. Chapple, Sophie F. Whittall, Peter W. Dillingham, Stephen R. Wing

**Affiliations:** ^1^ Department of Marine Science University of Otago Dunedin Otago New Zealand; ^2^ Department of Mathematics and Statistics University of Otago Dunedin Otago New Zealand

**Keywords:** fish ecology, individual specificity, kelp forest, life history recorders, niche breadth, stable isotope analysis

## Abstract

Fishes can undergo dramatic social and morphological changes throughout development that drive ontogenetic shifts in diet and habitat association. Measurements of trophic ontogeny at the individual level often complement population‐level assessments, detailing foraging strategies that have underpinned long‐term growth and survival. Using stable isotope measurements from muscle and eye lenses, we modeled size‐based patterns in basal resource use and trophic position across multiple levels of organization for three New Zealand reef fishes ( *Notolabrus fucicola* , *Odax pullus,* and *Parapercis colias* ). From lens‐derived data series, we were able to estimate trends and variability in lifetime trophic ontogeny of each species, as well as size‐structured changes in breadth and interspecific overlap of resource use. For adults, broadly similar trophic shifts were reflected in isotopic composition of both muscle tissue and lens layers, with subtle differences between tissues for some combinations of species and ecological metric. Critically, only samples from eye lenses yielded estimates of resource use that supported early growth. Specifically, our measurements suggested heightened reliance on macroalgal food webs during post‐settlement dispersal of two carnivores ( *N. fucicola* , *P. colias* ), as well as variable peaks in omnivory at small sizes for *O. pullus* , a primary herbivore. Trophic shifts modeled from eye lenses of carnivorous species were generally similar throughout early development, but highly inconsistent among juvenile *O. pullus* . Analyses of eye lenses also yielded evidence of trophic breadth contraction around size‐at‐maturity of all three species, coincident with apparent differentiation of adult resource use between sampled carnivores. Finally, in addition to high‐resolution assessments of trophic ontogeny, we provide analytic considerations that may strengthen use of eye lenses for investigation of fish life history, particularly through examination of calibration assumptions in a novel system.

## Graphical abstract

Here, we describe the results of stable isotope analysis using sequentially deposited layers from fish eye lenses to model lifetime trophic shifts of three New Zealand kelp forest species: *Notolabrus fucicola* , *Odax pullus* , and *Parapercis colias* . Using eye lenses, we identified size‐structured trends and patterns of variability in estimates of basal resource use and trophic position at individual, population, and community scales. Compared to muscle tissue, eye lens isotopic records provided higher resolution and more comprehensive information regarding trophic development, particularly during early life history. In addition to detailed discussion of trophic ontogeny in these three fishes, we provide novel considerations regarding key assumptions for reconstructing ecological information from analysis of eye lens tissue. Photo credit: Steve Wing.

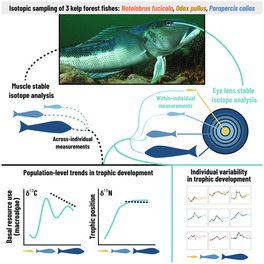

## 1 Introduction

In animal populations where diet or habitat associations change throughout development, careful descriptions of size‐structured resource use are essential for predicting the drivers and ecological consequences of demographic change (Werner and Gilliam 1984; Nakazawa 2015; Sánchez‐Hernández et al. 2019). Estimating individual variability in ontogenetic resource shifts can be equally important as measuring species‐level trends, providing quantifications of trophic plasticity that can confer resilience against environmental disturbance (Takimoto 2003; De Roos and Persson 2013). Growth‐related patterns in consumer resource use can be assessed at the individual level via sequential monitoring or at the population level via contemporaneous observation across multiple life phases (Werner and Gilliam 1984; Kim et al. 2012; Stallings et al. 2023). Both methods offer information regarding trophic ontogeny but also have approach‐specific limitations. For instance, changes in individual resource use over time can be difficult to measure in long‐lived species, while population‐level sampling can be biased towards common life stages that are convenient to observe or collect (Turner Tomaszewicz et al. 2017; Brady et al. 2020). Together, population‐ and individual‐level estimates of size‐structured resource use offer cumulative insights that warrant mutual consideration in the study of animal life history. However, synthesizing analyses of trophic ontogeny across scales first requires thoughtful comparison of approach‐specific findings, helping to parse ecological signal from methodological noise (Wittemyer et al. 2009).

Environmental tracers within animal tissues, such as stable isotopes, integrate information regarding diet or habitat use at timescales determined by tissue turnover period, often days or months (Post 2002; Layman et al. 2012). In discretely layered and metabolically inert tissues, sequential sampling of stable isotopes can yield refined descriptions of size‐structured resource use that span an individual's complete lifetime (Wittemyer et al. 2009; Tzadik et al. 2017). Individual‐level isotopic chronologies can usefully complement population‐level analyses of trophic ontogeny, providing high‐resolution information from relatively few specimens and life stages that are difficult to sample (Kim et al. 2012; Turner Tomaszewicz et al. 2017; Vecchio and Peebles 2022). In fish, stable isotope analysis is commonly performed using muscle tissue samples, often assumed to reflect composition of assimilated prey over the 1–3 months preceding collection (Trueman et al. 2005; Ankjærø et al. 2012). However, isotopic turnover rates of fish muscle vary by species and environment, and may extend towards annual time scales when somatic growth is slow (Suring and Wing 2009; Vander Zanden et al. 2015). Still, isotopic measurements from fish muscle tissue are usually consulted to provide a snapshot of ‘recent’ resource use, with population‐level trends captured by sampling across multiple individuals.

In the last decade, stable isotope records in fish eye lenses have been investigated as a promising tool for extending trophic histories throughout individual lifetimes (Wallace et al. 2014; Quaeck‐Davies et al. 2018; Bell‐Tilcock et al. 2021). Eye lenses grow via sequential deposition of concentric laminae which undergo a form of apoptosis and become metabolically inert, preserving isotopic information that can be recovered via ordered subsampling (Dahm et al. 2007; Wallace et al. 2014; Kuntz et al. 2025). In particular, the protein‐rich composition of eye lenses facilitates diet reconstruction using organic stable isotopes compared to alternate sequentially‐deposited tissues (i.e., scales, otoliths), where organic matter is sparse and tightly bound in inorganic matrices (Tzadik et al. 2017; Stounberg et al. 2022). Trophic metrics can be derived from isotopic measurements through mixing models that relate composition of consumer and producer tissues, often using ^13^C:^12^C to assess basal resource use and ^15^N:^14^N to estimate trophic position (Post 2002; Fry 2006; Phillips et al. 2014). Both C and N are abundant in fish eye lenses even at very small sizes, in some settings providing sufficient material for characterization of maternal provisioning or post‐larval settlement habitats (Simpson et al. 2019; Young et al. 2022; Rosinski et al. 2023). Eye lens isotopic analysis can thus substantially expand the timescale of inference provided by sampling of muscle or other metabolically active tissues, supporting comprehensive investigation of stage‐structured diet, movement, and trophic overlap among species (e.g., Kurth et al. 2019; Curtis et al. 2020; Vecchio and Peebles 2022; Diallo and Olden 2025).

Three key calibrations were originally outlined by Quaeck‐Davies et al. (2018) to maximize ecological utility of eye lens isotopic analysis: (1) converting lens radius to fish length through development of lens growth models; (2) accounting for isotopic fractionation between dietary sources and lens material through comparison with a reference tissue; and (3) validation of lens‐derived trophic chronologies through comparison with established patterns of resource use. These calibrations were recommended to be applied and tested in a diverse range of taxa and systems, building understanding of the effects of fish morphology, trophic guild, and environment on lens development and preservation. Subsequent studies have consistently reported close isometric (or slightly allometric) relationships between lens diameter and fish length in multiple species, allowing estimation of fish size during formation of inner lens material (Curtis et al. 2020; Vecchio et al. 2021; Bastos et al. 2024). However, methods for modeling lens growth have differed with regard to exclusion of pre‐apoptotic laminae (PAL), the most recently deposited material that typically constitutes the outer 30% of whole eye lenses (Kuntz et al. 2025). PAL removal prior to size calibration has been recommended based on observations of clearer isometry between hardened lens diameter and fish length (Leifsdottir and Campana 2023), measurement difficulty due to high viscosity (Simpson et al. 2019), and suspected metabolic activity in outer lens material that violates the assumption of isotopic preservation (Faletti and Stallings 2021). Although unknown, the within‐lens position where the isotopic record becomes essentially fixed is likely located inside the PAL, near the threshold where cell denucleation is largely complete (~90% whole lens radius; Schartau et al. 2009; Kröger 2013). If true, this would introduce a presently unquantified bias to lens growth models that assumed isotopic fixation at the leading lens edge (by including PAL in size calibrations) or interior transition between PAL and hardened lens (by excluding PAL from size calibrations). Specifically, whole‐lens calibrations might underestimate fish size at isotopic fixation for a given lens radius by ignoring metabolic turnover within outer PAL, while calibrations that exclude PAL would overestimate size at fixation if any fraction of PAL were isotopically inert. Outputs of competing lens growth models have never been explicitly compared in the same system, limiting the ability of researchers to consider analytic consequences of various approaches for fish size reconstruction.

Changes in isotopic ratios during metabolic transfer from diet items to lens deposits (discrimination factors) must also be addressed to derive robust trophic information from isotopic measurements of lens tissue (Post 2002; Phillips et al. 2014). Based on comparison with muscle (a well‐studied reference tissue), the magnitude of diet‐lens isotopic discrimination may differ within and among species, sometimes to an extent that could drive substantial variation in mixing model outputs (Quaeck‐Davies et al. 2018; Faletti and Stallings 2021; Bastos et al. 2024). In some cases, isotopic offsets between muscle and lens tissues may even be associated with body size, which might systematically undermine the reliability of lens‐based trophic chronologies if unadjusted (Young et al. 2022; Diallo and Olden 2025). Furthermore, isotopic discrimination can be affected by dietary protein quality and nitrogen assimilation rates, requiring particular correction for trophic modeling in distinct feeding guilds such as herbivores (Mill et al. 2007; Busst and Britton 2016). Despite their potential influence on resource‐use reconstructions, relationships between diet, size, and relative isotopic composition of lens and muscle tissue remain poorly understood, especially in non‐carnivorous fish species.

For the present study, we modeled chronologies of basal resource reliance and trophic position using isotopic records from the eye lenses of three kelp forest fishes in Aotearoa New Zealand. Specifically, we first analyzed the isotopic composition of muscle tissue and eye lenses from three species spanning a range of adult diets: rāwaru blue cod ( *Parapercis colias* ), tāngāngā banded wrasse ( *Notolabrus fucicola* ), and mararī butterfish ( *Odax pullus* ). We then compared individual‐level trophic ontogenies from eye lenses to population‐level patterns from muscle samples, highlighting differences in ecological information yielded by isotopic analysis of each tissue. Using lens‐derived data, we further compared trends and variability in lifetime trophic estimates among all three species, referenced to existing knowledge regarding their ecology and life history. Finally, we present detailed consideration of calibrations that might improve the accuracy of ecological analyses using eye lenses, including a novel lens growth model that assumes mid‐PAL isotopic fixation along with size‐ and species‐specific assessment of isotopic discrimination between muscle and lens tissues.

## 2 Materials and Methods

### 2.1 Study Species

For our study, we collected specimens of *Notolabrus fucicola* , *Odax pullus* , and *Parapercis colias* , three large‐bodied fishes common to nearshore reefs of southeast New Zealand. In addition to being regionally abundant, ecological investigation of these species is warranted due to their influential interactions within kelp forests (Taylor and Schiel 2010; Wing and Jack 2014; Udy et al. 2019), fisheries value (especially *P. colias* ; Morrison et al. 2014; Durante et al. 2020), endemism to Australia and New Zealand, and status as taonga or treasured in Māori culture. Adult diet composition varies widely among our study species, including generalized carnivory in *P. colias* , specialized invertivory in *N. fucicola* , and primary herbivory on large brown macroalgae in *O. pullus* (Russell 1983; Morrison et al. 2014). Development of all three species features at least partial incidence of sex change from female to male during maturation, coinciding with broad shifts in behavior, morphology, and resource use (Denny and Schiel 2001; Beer and Wing 2013; Johnson et al. 2020). Finally, these species generally exhibit high site fidelity and feed at small spatial scales following recruitment into shallow kelp forests (Cole et al. 2000; Edgar et al. 2004; Morrison et al. 2014). Resident fishes are ideal targets for eye lens analysis, facilitating interpretation of isotopic fluctuations among exterior (more recently deposited) laminae as signal for dietary shifts within site‐specific food webs rather than movement among habitats (Curtis et al. 2020; Young et al. 2022; Vecchio and Peebles 2022). Conversely, isotopic trends measured from inner lens material, corresponding to fish sizes smaller than those commonly observed in shallow reefs, can be primarily interpreted as dietary changes during juvenile migration from settlement grounds (Vecchio and Peebles 2020; Faletti and Stallings 2021).

### 2.2 Fish Sampling

**FIGURE 1:**
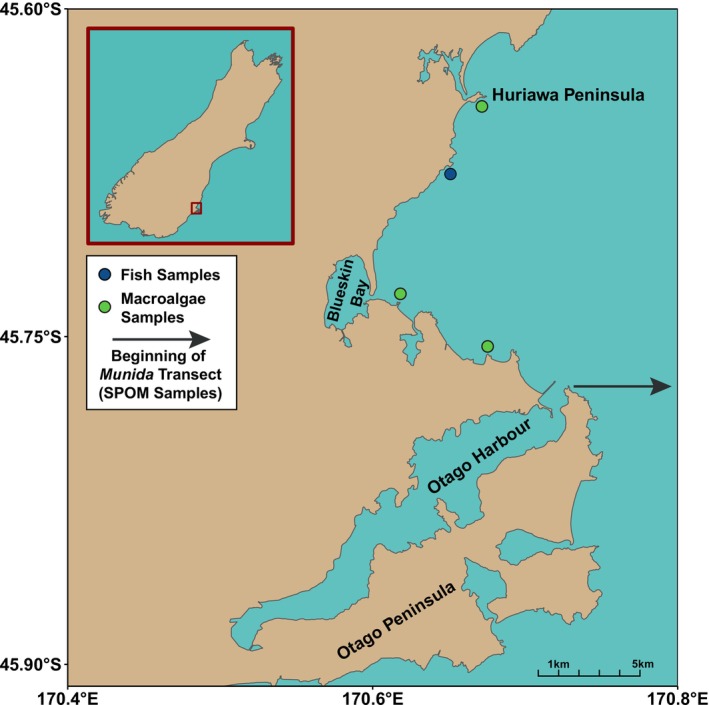
Map of study region, including collection sites for fishes (blue) and macroalgae samples (green). A more detailed map of SPOM sampling sites (stations 1–6, *Munida* transect) is available in Durante et al. (2021). Inset map of Te Wai Pounamu the South Island, with study region indicated in red.

We collected fish specimens from rocky reefs near Te Awa Koeo Brinn's Point off the south Otago coast in February 2022 (Figure 1). Reefs near the sampling site support diverse macroalgal communities including surface canopy‐forming *Macrocystis pyrifera* and other phaeophytes, notably *Carpophyllum* spp., *Undaria pinnatifida* and *Ecklonia radiata*. Individual *O. pullus* and *N. fucicola* were collected via spearfishing from rocky outcrops in 5–7 m depth. We gathered *P. colias* using baited traps set in 15 m depth. For all species, we retained fish across the size range observed during sampling (Table 1).

**TABLE 1:** Summary of sampling efforts, distributions of total length (cm) for study species, and isotopic values of muscle tissue (‰).

Species	Fish sampled	Min. length	Max. length	Mean length	δ^13^C mean (±SD)	δ^15^N mean (±SD)	Lenses sampled
*Notolabrus fucicola*	25	13.0	35.9	25.7	−18.8 ± 0.8	14.7 ± 0.2	25
*Odax pullus*	32	19.8	44.0	33.2	−16.8 ± 0.6	11.6 ± 0.3	19
*Parapercis colias*	40	18.3	38.9	27.9	−18.6 ± 0.5	13.9 ± 0.4	19

Fishes were euthanized via pithing and stored on ice for transportation to the Portobello Marine Laboratory, where they were frozen whole. After thawing, we measured total length (TL, cm) and wet mass (g) using a benchtop balance (±0.1 g). We then collected and froze a clean sample of dorsal musculature (~1 cm^3^) at −20°C for isotopic analysis. Whole fish eyes were extracted, wrapped in aluminium foil and refrozen intact at −20°C.

### 2.3 Lens Delamination

Terminology regarding eye lenses varies, so we first clarify our usage before describing our dissection protocol. We use ‘layer’ to mean a section of lens tissue of approximately equal thickness removed during delamination, which we distinguish from an individual lamina, or the tissue accreted during a single growth phase (Wallace et al. 2014). Most sampled layers probably consisted of several adhered laminae. We use PAL to refer to the large portion of outermost, semi‐fluid lens material, which is distinct from hardened cortical lens and assumed to be at least partially metabolically active (Schartau et al. 2009; Kuntz et al. 2025). PAL are sticky and difficult to remove cleanly from hardened lens material without dissolution or abrasion, both of which preclude retention for isotopic analysis (Simpson et al. 2019; Leifsdottir and Campana 2023). We therefore sampled PAL and hardened lens layers as a continuum along a whole‐lens transect, paired with notes regarding tissue consistency. Lens samples spanning the “transition zone” likely contained a mixture of PAL and underlying material, either because shards of hardened lens were embedded in PAL or remnants of PAL were adhered to the outermost hardened lens. Finally, we refer to data series retrieved from lenses as “chronologies”, a common shorthand in studies of sequentially deposited tissues even when patterns are not time‐resolved (Tzadik et al. 2017; Stounberg et al. 2022).

We performed eye lens analysis on a subset of individuals spanning the sampled size range for each species, except for *N. fucicola* for which all available lenses were analyzed (Table 1). Prior to lens extraction, a single eye for each fish was chosen randomly between left and right eyes (assumed to be isotopically equivalent; Wallace et al. 2014; Young et al. 2022) and defrosted at room temperature. To delaminate extracted eye lenses, we used a protocol modeled on previous applications in similarly sized fishes (Wallace et al. 2014; Curtis et al. 2020; Vecchio et al. 2021). Specifically, we removed the lens capsule using fine‐tipped stainless‐steel forceps and lightly rinsed remaining material with deionized (DI) water in a glass petri dish. We then measured maximum lens diameter at the widest point to the nearest 0.01 mm using Vernier callipers (Kincrome K11105). Following measurement, we air dried lenses until the PAL was firm enough to retain its shape when manipulated. We then peeled PAL away from underlying cortical material and measured remaining lens diameter. Tools and dishes were wiped clean with ethanol and lint‐free tissues between removal of each layer. We repeated this process to extract hardened lens layers until the remaining lens reached ~0.5–0.7 mm diameter, the minimum size that yields sufficient material for reliable measurement of ^15^N:^14^N. During delamination, whenever the lens dried to the point of becoming brittle, it was submerged in DI water until it regained pliability, usually for 5–10 s.

### 2.4 Stable Isotope Analysis

Prior to isotopic analysis, muscle samples were lyophilized at −55°C for 48 h and homogenized using a ceramic mortar and pestle. During sample preparation, 0.5–0.7 mg of muscle tissue was loaded into tin capsules and weighed using a benchtop microbalance (Precisia EP 225SM‐DR, ±0.01 mg). Following delamination, lens layers were stored in a polypropylene 96‐well microplate and air dried overnight in a laminar flow hood. Larger layers (> 0.7 mg) were coarsely crushed and mixed prior to weighing, with a 0.5–0.7 mg subsample submitted for analysis. Smaller layers (0.19–0.7 mg) were weighed and tinned in their entirety. Where possible, the smallest layers (< 0.19 mg) were combined with approximately equal mass of an adjacent layer to achieve sufficient mass for isotopic analysis. Stable isotope ratios were measured at the Earth Sciences New Zealand (formerly GNS) Stable Isotope Laboratory using a continuous flow EA‐IRMS system consisting of a Eurovector elemental analyser coupled with an Isoprime isotope ratio mass spectrometer. Using an internal Leucine standard, ^13^C:^12^C ratios in samples were normalized to the VPDB scale, while ^15^N:^14^N sample ratios were normalized to the AT‐air scale. Isotopic measurements are hereafter described using standard δ notation and per mille units (‰). Instrument analytical precision was 0.3‰ for δ^15^N and 0.2‰ for δ^13^C.

### 2.5 Data Analysis

#### 2.5.1 Isotopic Mixing Model

Raw stable isotope values were converted to trophic estimates by relating isotopic composition of fish tissues to average measurements from two basal resource pools: suspended particulate organic matter (SPOM) and palatable brown and green macroalgae. SPOM samples were collected in January 2018 from stations 1–6 along the *Munida* transect, 15–45 km offshore of the Otago Peninsula (*n* = 7; Figure 1; Durante et al. 2021). Seawater for SPOM filtration was collected from the surface (1.5 m depth) or chlorophyll maximum layer (20 m depth). Macroalgal blades were sampled between 2018 and 2022 from three shallow (< 10 m depth) rocky reefs between the Huriawa Peninsula and Otago Harbour mouth (*n* = 109; Figure 1; McCarthy et al. 2024). Additional macroalgal and SPOM collections from the Otago Harbour were initially considered for inclusion, especially to provide SPOM samples from inshore reefs. Although average δ^13^C values of Harbour SPOM and macroalgae were within 1‰ of measurements from other sampling areas, δ^15^N values were on average > 2‰ higher, likely reflecting proximity to population centers and anthropogenic inputs (Schlieman et al. 2022). We therefore opted to exclude Harbour collections from our model, though these still provided reassurance that SPOM δ^13^C values from offshore sampling were similar to coastal measurements. Detailed protocols for collection, processing, and isotopic analysis of source pool samples are provided in the methods of respective publications (SPOM: Durante et al. 2021; macroalgae: McCarthy 2025).

For our mixing model, we entered consumer and source‐pool isotopic values into mass‐balance equations (Post 2002). Using δ^13^C values, we calculated preliminary estimates of proportional producer inputs as:

(1) $$P_{\text{SPOM}}=\frac{\delta^{13}C_{\text{Sample}}-\delta^{13}C_{\text{Macro}}}{\delta^{13}C_{\text{SPOM}}-\delta^{13}C_{\text{Macro}}}$$

where consumer isotopic ratios are measured for individual samples and source isotopic ratios represent the averages for each producer. We then calculated a preliminary estimate of trophic position (*T*) as:

(2) $$T_{\text{init}}=1+\frac{\delta^{15}N_{\text{Sample}}-\left(P_{\text{SPOM}}\left(\delta^{15}N_{\text{SPOM}}\right)+P_{\text{Macro}}\left(\delta^{15}N_{\text{Macro}}\right)\right)}{3.4}$$

where 3.4 is the expected fractionation of δ^15^N per trophic level (Post 2002). Although alternate trophic enrichment factors have been presented for carnivorous fishes (McCutchan et al. 2003), we selected 3.4 as a commonly applied global average that would better accommodate the herbivorous *O. pullus* . An enrichment factor of 3.4 for δ^15^N has also been shown to yield reasonable descriptions of *P. colias* trophic position, matching estimates from stomach content analysis (Kolodzey et al. 2023). Using *T*_init_, we adjusted our estimate of basal resource mixture for expected fractionation of δ^13^C with changes in trophic position (0.4‰/trophic level; Post 2002) using:

(3) $$\delta^{13}C_{\mathrm{Adj}}=\delta^{13}C_{\text{Sample}}-0.4T_{\text{Init}}$$

then reapplying Equation (1) with δ^13^C_Adj_ in place of δ^13^C_Sample_. Finally, we recalculated trophic position using Equation (2) and adjusted estimates of basal resource mixture (from Equation 3). We iterated this adjustment cycle twice, achieving stable estimates of *P*_SPOM_, *P*_Macro_, and *T* for each sample. As outputs from a two‐source mixing model, *P*_SPOM_ and *P*_Macro_ were truncated at 1 and always summed to 1 (100%) for a given sample.

#### 2.5.2 Eye Lens Calibrations

To estimate fish size corresponding to material at a given lens radius, we first recorded the radial midpoint of each layer, defined as the average radial distance from the lens center (Wallace et al. 2014; Vecchio et al. 2021). If layers were combined during sample preparation, their radial midpoints were averaged for subsequent analyses. Two common approaches for relating lens size to fish length rely on measurements of whole lens diameter (including PAL) or hardened lens diameter (excluding PAL) as reference points for calibration (e.g., Quaeck‐Davies et al. 2018; Faletti and Stallings 2021). To model the likely possibility that isotopic turnover ceases between these two boundaries (Schartau et al. 2009; Kröger 2013; Kuntz et al. 2025), we related fish length to lens radius using a calibration point (*C*) within the PAL, calculated as:

(4) $$H:W_{s}=\text{Mean}\left(\frac{R_{\text{Hard}}}{R_{\text{Whole}}}\right)$$

(5) $${\text{C}}_{i}=1.12\left(H:W_{s}\times R_{\text{Whole},i,s}\right)$$

where *H*:*W*_s_ is the average proportion of hardened lens within whole lenses for each species (s), *R*_Whole_ is whole lens radius for each individual (i) of species s, and 1.12 is a constant that adjusts for average proportional thickness of hardened lens layers (Figures S1–S3). Radial distance from the lens center at C approximates the boundary of the layer being formed within inner PAL at time of sampling, which would have become distinguished along a new transition between PAL and hardened lens had growth continued.

Relationships between fish length and lens size are often approximately linear in larger individuals, but may also exhibit nonlinear allometry at smaller sizes (Simpson et al. 2019; Curtis et al. 2020; Vecchio et al. 2021). To model lens growth across the sampled length range for each species, we first identified the best‐fit relationship between lens radius at C and fish total length among three options: a linear, power‐curve, and log‐quadratic model (see the Supporting Information for formulae and model selection criteria). None of the best‐fit lens growth models could be extrapolated to predict lens radius at fish lengths below the sampled size range while satisfying three realistic criteria: an intercept near (0,0), monotonic lens growth, and positive values of estimated total length (ETL). We therefore estimated unobserved lens growth using a linear interpolation between (0,0) and the smallest calibration point for each species. Although lenses likely grow in some non‐linear manner with fish size in early life, the bias between reasonable alternatives and our linear approximation should be qualitatively minor in the context of broad conclusions regarding trophic ontogeny (Figure S4).

From the best‐fit models, ETL for outermost lens samples exceeded observed fish length if C was interior to the PAL radial midpoint, which was true in most cases. For each chronology, we therefore set maximum ETL to observed total length. In two individuals where multiple samples were collected from radial positions exceeding C, exterior isotopic values were averaged before further analyses. For size calibrations in *P. colias* , 55 additional measurements were included from individuals collected in the same region for a different study (Curtis 2025, Chapter 3). Data from these specimens were not included in any other analyses.

To adjust for potential differences in diet‐tissue discrimination factors prior to trophic modeling, we first measured the offset in δ^13^C and δ^15^N values between PAL and muscle tissue for each individual (Quaeck‐Davies et al. 2018; Young et al. 2022; Bastos et al. 2024). We then applied mean species‐specific corrections to isotopic values measured across all lens layers. Individuals were excluded from both calibration calculations (size and isotopic) if PAL could not be measured or retained, usually due to dissolution during rinsing.

#### 2.5.3 Statistical Analyses

Application of an average isotopic adjustment across lens transects assumes interior lens layers were once PAL and featured similar, size‐invariant isotopic offsets from diet and muscle tissue. We examined this assumption using linear regression to quantify relationships between lens‐muscle isotopic offsets and fish length for each isotope‐species pair. Lens‐derived trophic chronologies typically assume complex non‐linear forms that are difficult to anticipate ahead of analysis. Following Simpson et al. (2019), we therefore applied GAMMs to assess trends in trophic metrics (*P*_Macro_ or *T*) estimated from lens isotopic values with changes in ETL. GAMMs can accommodate a wide array of non‐linear relationships without requiring predetermination of underlying patterns (Wood 2017). Relationships between observed fish length and trophic estimates from muscle isotopic values were analyzed using GAMs and linear models, with GAMs favored if: (1) there was strong statistical evidence for a fit of the modeled smoother to the data (95% CI for test statistic *χ* ^2^ excluding 0) and (2) ΔAIC_LM‐GAM_ >> 2 (Bozdogan 1987). We excluded muscle isotopic values that were both extreme (outside of mean ± 2.5 SD for each species‐isotope pair) and observed to qualitatively influence model fits. Models with *P*_Macro_ as a response variable (a continuous proportion bounded at (0,1)) were fit using a beta distribution for family and a logit link, while models using *T* as a response variable were assigned a Gaussian family and an identity link (Wood 2017).

For all GAMMs, we fit models using *mgcv* with the basis‐dimension parameter (*k*) set to 10, thin plate regression splines (‘tprs’) as the smoother basis, and restricted maximum likelihood (REML) as the smoother selection method (Wood 2017). GAMM fits were assessed using output from *mgcv* and *gratia* (Simpson 2024), particularly patterns in model residuals and relative values for estimated degrees of freedom (*edf*) and *k*. Finally, GAMMs for lens chronologies were modeled using a smoother for fish identity and a random‐effect smoother basis (‘re’) to account for autocorrelation. The resulting formulation resembles the *G* model described in Pedersen et al. (2019), and assesses the fit of a global smooth relationship with a random intercept for each lens chronology. Differences between population‐level trophic ontogenies from lens and muscle tissues were assessed based on overlap of 95% CIs visualized using *gratia* and *ggplot, respectively* (Wickham et al. 2019). Size‐structured deviations of individual chronologies from per‐species smooth fits were assessed quantitatively using GAMM CI width, as well as qualitatively using paired visualizations. Residuals between global trends and isolated chronologies can be interpreted as individual variability in trophic ontogeny, or alternate shifts in estimated resource use to the “average” conspecific at a given size.

Finally, we estimated lifetime patterns in resource‐use breadth for each species using SIBER to model the standard ellipse area (SEA_c_, adjusted for small sample sizes) of lens‐derived trophic metrics across chronologies (Jackson et al. 2011). We measured SEA_c_ within a 6 cm ETL window that was advanced in 1 cm iterations, the minimum bin width that provided sufficient data at each step (*n* ≥ 10). We also used SIBER to model continuous overlap in isotopic niche between *N. fucicola* and *P. colias* following the same analytic framework. Proportional overlap was calculated as the ratio of overlapping to non‐overlapping areas of 95% ellipsoids for both species. Posterior estimates for SEA_c_ and isotopic niche overlap were calculated using default SIBER arguments (Jackson et al. 2011) and 90% credible intervals (HDI‐based cutoffs) were estimated using *bayestestR* (Makowski et al. 2019). For all analyses, strength of statistical evidence was determined based on effect sizes and confidence or credible intervals (relative to 0), and results are described using language adapted from Muff et al. (2022). All analyses were performed in R v4.2.2 (R Core Team 2024).

## 3 Results

### 3.1 Calibrations

**FIGURE 2:**
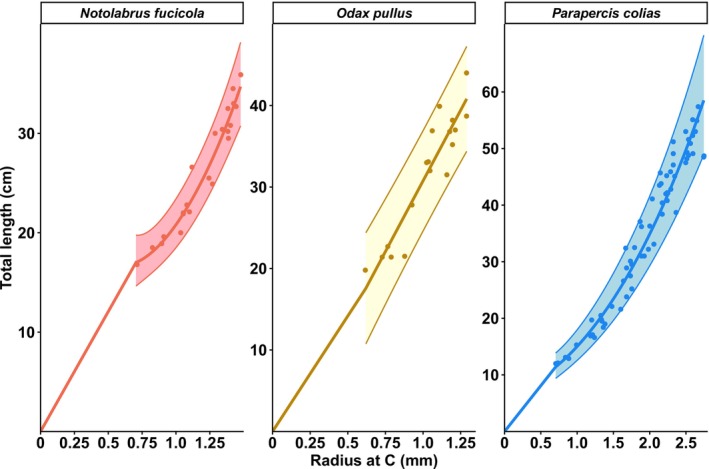
Predicted fits (lines) of lens radius at calibration point C (Equations 4 and 5) against fish length. Scales vary by species. Trendlines and 95% prediction intervals were generated using *investr* (Greenwell and Kabban 2014) across the observed data range. Below the smallest calibration point, a linear interpolation through the intercept is shown without uncertainty. A log‐quadratic model of lens growth was used for *N. fucicola* and *P. colias* , while linear lens growth was modeled for *O. pullus* (Table 2; Table S1). In all cases, best‐fit models indicate tight relationships between lens and body size.

Within the range of observed data, lens radius at calibration point C was tightly related to length in all three species, with linear lens growth modeled for *O. pullus* and non‐linear lens growth modeled for *N. fucicola* and *P. colias* (Figure 2, Table 2; Figure S4, Table S1). Although lengths estimated from lens measurements (ETL) are hereafter presented using regression averages without uncertainty, these are only reliable to approximately ±3 cm for *N. fucicola* , ±6 cm for *O. pullus* , and ±4 cm for *P. colias* based on prediction interval width for corresponding models (Figure 2).

**TABLE 2:** Summary of parameters for fitted relationships between fish length and eye lens radius, visualized in Figure 2. See the Supporting Information for details regarding regression model formulae (Equations S1–S4) and selection criteria (Table S1).

Species	Regression	Coefficient	Estimate	95% CI	*R* ^2^
*Notolabrus fucicola*	Log‐Quadratic	*β*_0_	3.04	3.00, 3.07	
		*β*_1_	0.92	0.78, 1.07	0.946
		*β*_2_	0.99	0.42, 1.56	
*Odax pullus*	Linear	*β*_0_	−3.94	−11.47, 3.59	0.858
		*β*_1_	34.69	27.50, 41.89	
*Parapercis colias*	Log‐Quadratic	*β*_0_	2.71	2.67, 2.75	0.941
		*β*_1_	0.93	0.79, 1.07	
		*β*_2_	0.41	0.26, 0.57	

**FIGURE 3:**
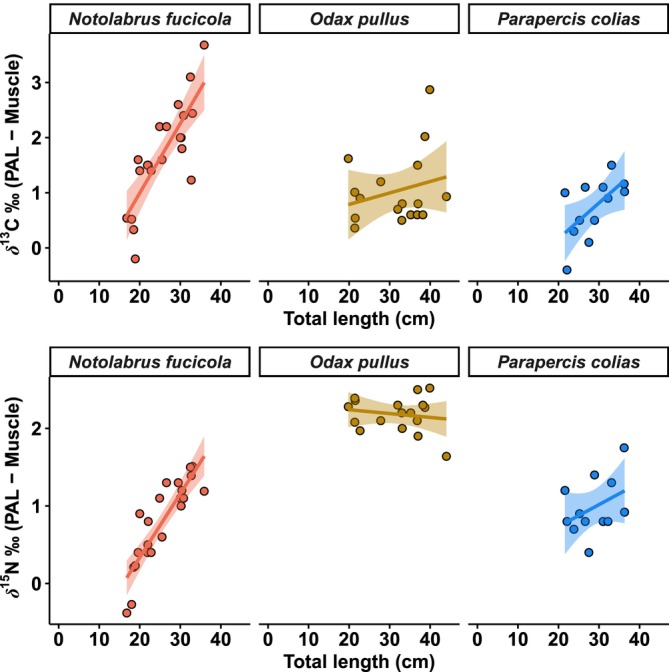
Fits (with 95% CIs) for linear regressions of isotopic offsets between outermost lens material (pre‐apoptotic laminae, PAL) and muscle tissue against total length for each study species. Data distributions and model fits are summarized in Table 3. Isotopic offsets were not clearly related to body size in *O. pullus* or *P. colias* , but were strongly associated with length in *N. fucicola* .

The magnitude and consistency of isotopic offsets between PAL and paired muscle tissue varied by species‐isotope pair (Figure 3, Table 3). In most cases, average PAL isotopic values were ~1‰ higher than measured in paired muscle tissue. However, δ^13^C_PAL‐Muscle_ offsets in *N. fucicola* were slightly larger (though more variable) and δ^15^N_PAL‐Muscle_ consistently exceeded 2‰ in *O. pullus* . Isotopic offsets were unrelated to length in *O. pullus* , while there was moderate evidence for a positive relationship between δ^13^C_PAL‐Muscle_ and length of *P. colias* (Figure 3, Table 3). Conversely, both δ^13^C_PAL‐Muscle_ and δ^15^N_PAL‐Muscle_ in *N. fucicola* were strongly associated with total length (*r* ^2^ > 0.65).

**TABLE 3:** Summary statistics for offsets between isotopic values (‰) measured in outermost lens material (pre‐apoptotic laminae, PAL) and muscle tissue, as well as values of *t* and *r* ^2^ from linear models describing relationships of isotopic offsets and fish size (visualized in Figure 3).

Species	*n*	δ^13^C (PAL–Musc.)			Size LM		δ^15^N (PAL–Musc.)			Size LM	
		Mean	Med.	SD	*t*	*r* ^2^	Mean	Med.	SD	*t*	*r* ^2^
*Notolabrus fucicola*	22	1.70	1.60	0.90	6.19	0.66	0.79	0.95	0.55	7.96	0.76
*Odax pullus*	17	1.03	0.80	0.65	0.99	0.06	2.18	2.20	0.23	−0.67	0.03
*Parapercis colias*	12	0.73	0.95	0.54	2.47	0.38	0.98	0.85	0.37	1.36	0.16

Isotopic values from muscle tissue were well constrained within the bounds of source pools in our mixing model (Figure S5). The only exceptions were 20 (of 32) muscle samples and 31 (of 122) lens samples from *O. pullus* with isotopic measurements outside of the average mixing space. For these samples, *P*_Macro_ could be confidently truncated from values exceeding 100% to equal exactly 100%, a reasonable estimate given the primarily herbivorous diet of the species (Clements and Choat 1993).

### 3.2 Trophic Patterns

**FIGURE 4:**
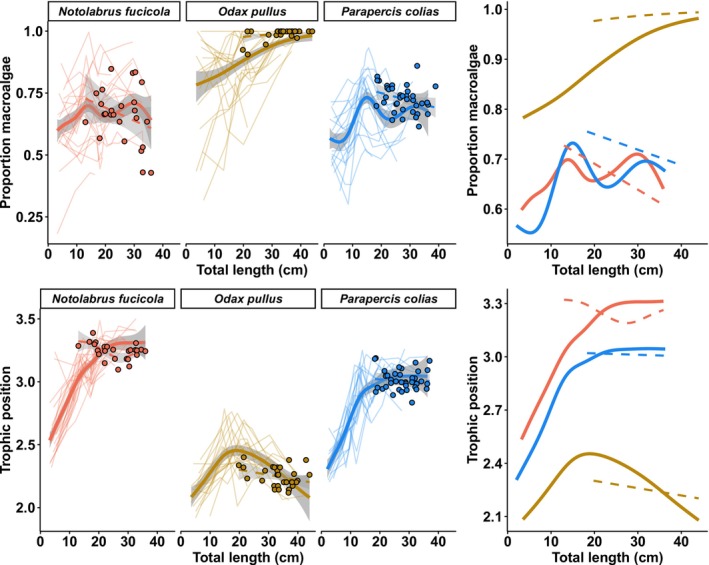
Trends and 95% CIs for fits of smooth relationships (via GAMMs) or linear models between trophic estimates and fish size (observed for muscle tissue, estimated for lens chronologies) using isotopic measurements of eye lens layers (solid) or muscle tissue (dashed). Constituent data are visualized as thinner lines (eye lens) or points (muscle). CIs were plotted using *ggplot* for muscle samples and *gratia* for eye lenses. CI overlaps indicate similar trophic estimates from the two tissues at associated fish lengths.

Isotopic values from muscle tissue provided species‐ and metric‐dependent evidence for changes in food web position across the sampled size range (Figure 4; Table S2). For *N. fucicola* , modeled macroalgal assimilation was not clearly associated with size, while estimated trophic position oscillated by a small amount. In *O. pullus* , muscle isotopic values suggested a small proportional input of SPOM‐derived productivity in individuals 20–30 cm TL, followed by saturation at an entirely macroalgae‐derived diet in larger fish. These coincided with a weakly defined and subtle decrease in muscle‐based estimates of *O. pullus* trophic position with size. Finally, isotopic measurements of *P. colias* muscle tissue yielded moderate evidence for a subtle decline in macroalgal reliance with growth, but size‐invariant estimates of trophic position.

In all three species, GAMMs provided strong statistical evidence for nonlinear changes in lens‐derived trophic metrics throughout growth (Figure 4, Table 4). For *N. fucicola* and *P. colias* , global smooth fits for modeled *P*_Macro_ oscillated with peaks at ~15 and ~30 cm ETL, while trophic position was estimated to sharply increase before stabilizing at ~23 cm ETL. For *O. pullus* , average lens‐based estimates of macroalgal contribution steadily increased before stabilizing at values consistent with total herbivory, with modeled trophic position changing parabolically and peaking at an average ~18 cm ETL.

**TABLE 4:** Summary statistics describing smooth fits (from GAMMs) of lens‐derived trophic estimates (*P*_Macro_: basal resource mixture; trophic position: *T*) against estimated fish length at isotopic fixation of lens tissue (ETL). Estimated degrees of freedom (*edf*) describe the degree of non‐linearity of the global smooth fit (with 1 corresponding to a linear fit), and the goodness‐of‐fit metric DevExp indicates the proportional deviance explained by each model. GAMM fits are visualized in Figure 4.

Species	Response	edf	*χ* ^2^	DevExp
*Notolabrus fucicola*	*P*_Macro_	5.34	22.37	0.787
	*T*	4.41	590.23	0.848
*Odax pullus*	*P*_Macro_	2.10	58.99	0.672
	*T*	4.20	90.64	0.562
*Parapercis colias*	*P*_Macro_	6.29	105.14	0.782
	*T*	4.75	386.82	0.800

Overlap between muscle‐ and lens‐derived assessments of resource‐use ontogeny varied by trophic metric and species (Figure 4). In all cases, eye lenses provided estimates of resource use at smaller sizes than possible using available muscle tissue samples, recording information from approximately days or weeks following settlement (2–3 cm ETL; Welsford et al. 2004). At fish sizes represented in both datasets, muscle‐derived estimates of basal resource use suggested higher and less size‐structured reliance on macroalgae for *O. pullus* and *P. colias* , with between‐tissue differences narrowing at larger sizes. For *N. fucicola* , CIs from muscle‐ and lens‐based trophic models mostly overlapped across the shared size range. Between‐tissue differences in estimated trophic position were more similar than modeled for *P*_Macro_, most notably in *P. colias* . At the community level, among‐species offsets in modeled trophic position were consistent between tissues, as were estimates of heightened *P*_Macro_ for *O. pullus* relative to sampled carnivores (Figure 4; Figure S5). Conversely, analysis of muscle isotopic composition suggested a weak trend of higher macroalgal reliance in *P. colias* than *N. fucicola* , which was not conserved in lens‐based models.

**FIGURE 5:**
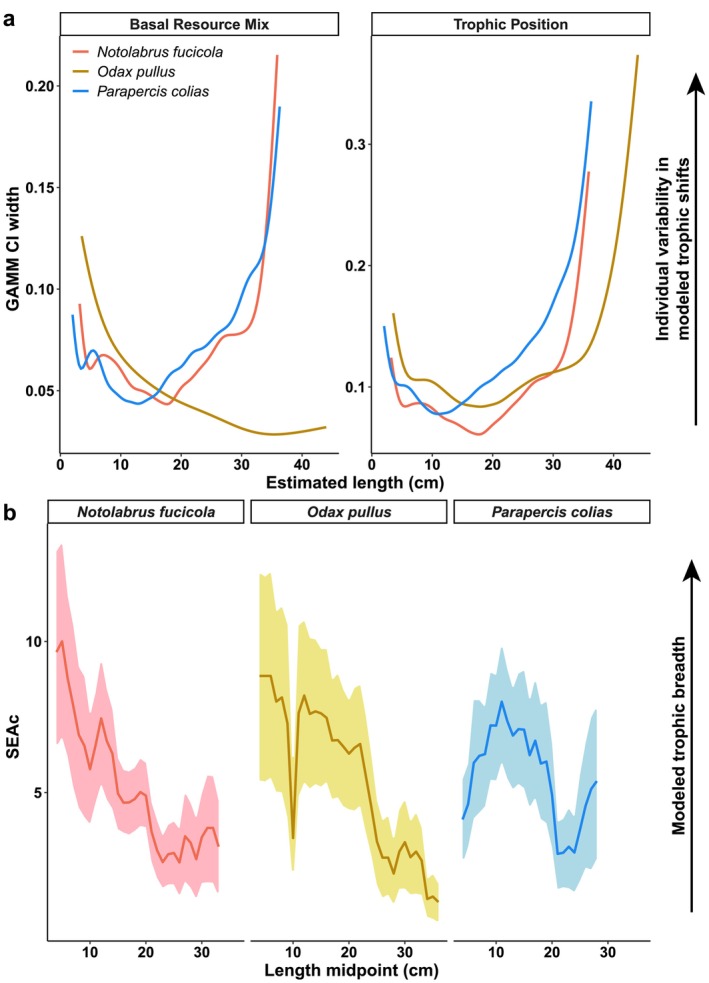
(a) Width of 95% CIs around GAMM fits assessing relationships between trophic estimates and modeled length‐at‐fixation of eye lens isotopic records (ETL), visualized in Figure 4. Higher values indicate greater uncertainty around fitted trophic chronologies, or lower among‐individual consistency in slopes of modeled trophic shifts. (b) Standard ellipse area (SEA_c_) and 90% credible interval of estimated trophic breadth across lens chronologies. Ellipses were measured within a 6 cm moving window, and are plotted against window midpoint (ETL). Higher values indicate broader dispersion of trophic estimates across the subsetted size range.

Lens‐derived trophic chronologies were moderately to strongly coherent among individuals of each species, though least consistent in *O. pullus* (based on deviance explained, Table 4). In *N. fucicola* and *P. colias* , GAMM CI width (reflecting among‐individual variation in modeled trophic slopes) was lowest between 10 and 20 cm ETL and substantially increased at larger sizes (Figure 5a). Conversely, modeled basal resource chronologies in *O. pullus* were highly variable in early life but coherent at larger sizes, with sustained estimates of > 90% macroalgal contribution above 30 cm ETL. Spikes in GAMM CI width for the largest *P. colias* and *N. fucicola* were likely due to data limitations, and do not unambiguously suggest dramatic increases in among‐individual variability in late trophic ontogeny (although sparse but consistent data yielded high confidence in modeled *P*_Macro_ trajectories for large *O. pullus* ; Figure 5a).

For *N. fucicola* chronologies, individual deviations from average GAMM fits were mostly small and qualitatively inconsistent, except for two large positive residuals in trophic position modeled from innermost lens samples (Figure S6). In *P. colias* , a few *P*_Macro_ chronologies sharply deviated from model averages in the innermost or outermost lens layers. Additionally, early peaks in both trophic metrics varied in magnitude and timing among individual *P. colias* , usually expressed around 10–15 cm ETL where present. Trajectories of *P*_Macro_ modeled in *O. pullus* below ~17 cm ETL were the most variable of any species‐metric combination, with inconsistent and occasionally steep rates of change (Figure S6). Almost all *O. pullus* chronologies exhibited a mid‐lens peak in modeled trophic position, followed by disparate paths of recession towards estimates of primary consumption (Figure S6).

**FIGURE 6:**
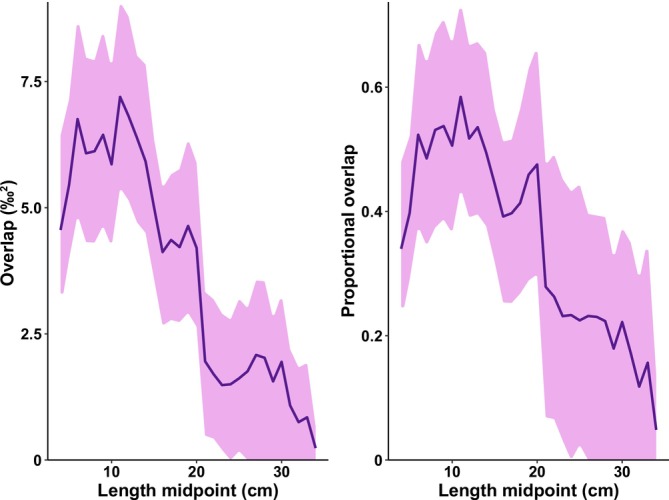
Trends and 90% credible intervals for changes in raw (‰^2^) and proportional estimates of isotopic niche overlap between *N. fucicola* and *P. colias* with modeled length‐at‐fixation of eye lens isotopic records (ETL). Overlap was measured within a 6 cm window of estimated fish length that was iteratively moved across eye lens chronologies, and generally decreases with growth.

Estimates of resource‐use breadth (SEA_c_) also varied markedly along aggregated lens transects (Figure 5b). Modeled trophic values in *N. fucicola* and *P. colias* were most dispersed at smaller sizes, with SEA_c_ declining to minima between 20 and 25 cm ETL. Trophic breadth estimates were similarly highest for *O. pullus* below 22 cm ETL (besides a distinguished local minimum at 10 cm ETL), then sharply contracted towards the lowest SEA_c_ values modeled among the three species. Finally, lens‐derived estimates of isotopic niche overlap between *N. fucicola* and *P. colias* were highest at smaller sizes, precipitously declined at 20 cm ETL, and reached a minimum for the largest size classes (Figure 6).

## 4 Discussion

In the present study, stable isotope measurements from layered eye lenses facilitated high‐resolution modeling of trophic ontogeny for three kelp forest fishes, providing particular insight regarding trends and variability in resource use during early life. Even at larger fish sizes, analysis of lens tissue yielded more detailed estimates of size‐structured trophic shifts than paired muscle samples, especially for basal resource use. In the two carnivorous study species, lens‐based estimates of macroalgal reliance peaked at 12–15 cm ETL, more strongly for *P. colias* than *N. fucicola* (Figure 4). A similar pattern measured in other fishes (the “carbon bump”) has been interpreted to reflect simultaneous onshore migration and enhanced feeding on benthic invertebrates in the months following settlement (Faletti and Stallings 2021; Vecchio and Peebles 2022). Post‐settlement dispersal from offshore to coastal areas is consistent with life history descriptions for *N. fucicola* and plausible for *P. colias* , which is thought to settle into deep‐water biogenic reefs (Wood and Probert 2013; Morrison et al. 2014). Our results suggest that reductions in macroalgal productivity might interrupt trophic development of juvenile *N. fucicola* and particularly *P. colias* , the effects of which could be investigated through comparison of lens‐derived resource use chronologies and individual fitness across life stages (*sensu* Post 2003).

Lifetime increases in estimated trophic position of *N. fucicola* and *P. colias* approximated parallel saturating growth curves, following a common trajectory observed in eye lenses of other carnivorous marine fishes (Figure 4; Curtis et al. 2020; Vecchio et al. 2021; Faletti and Stallings 2021). In fish, early adoption of a higher trophic position can lead to positive outcomes including increases in maximum size, growth rate, and energy storage compared with slowly developing individuals (Werner and Hall 1988; Post 2003). In our carnivorous study species, rapid trophic growth in early life appeared to be associated with enhanced consumption of macroalgae‐derived prey, based on coinciding peaks in both metrics (Figure S6). Therefore, availability of macroalgal resources for juvenile *N. fucicola* and *P. colias* might promote long‐term success by facilitating increases in trophic position, potentially underpinning associations between kelp forest foraging and heightened adult growth rates (Beer and Wing 2013). Lens‐ and muscle‐based trophic growth models for both species converged in fishes > 20 cm ETL, providing similar and broadly size‐invariant predictions for average trophic position. Our assessments of a stable trophic position at larger sizes match previous observations for *N. fucicola* and *P. colias* , which are thought to exhibit resident feeding habits that reflect local prey composition rather than consistent shifts in adult diet (Denny and Schiel 2001; Jiang and Carbines 2002; Rodgers and Wing 2008). Model averages for both species were ~0.5 trophic levels lower than some observations from other regions, though still reasonable for marine carnivores feeding primarily on small invertebrates (Davis and Wing 2012; Udy et al. 2019). Relatively low estimates of trophic position could indicate that our choice of 3.4 as a δ^15^N trophic enrichment factor was too high, and that future isotopic modeling in *P. colias* and *N. fucicola* should consider a more conservative value (possibly 2.3‰/trophic level; McCutchan et al. 2003). Regardless of whether our absolute estimates were slightly biased, relative trophic offsets among species or relationships with body size should be comparatively robust to enrichment factor uncertainty in sympatric fishes with similar diets (Post 2002; Suring and Wing 2009).

In contrast to the studied carnivores, lens‐based trophic models suggested that sampled *O. pullus* generally began life as a primary consumer, assimilated increasing proportions of animal material until sub‐adulthood, and fed on a narrow breadth of brown macroalgae as mature adults (Figure 4). Corroborating our results from eye lenses, isotopic assessments of *O. pullus* muscle tissue in other settings have indicated similar post‐juvenile declines in omnivory with growth (Johnson et al. 2020). Conversely, isotopic analysis of our own muscle samples did not yield clear relationships between *O. pullus* trophic position and size, potentially due to the relatively small number and length range of sampled individuals (a common limitation for opportunistic collections from wild populations). Although lens‐based trophic estimates for small *O. pullus* were highly variable and broadly inconsistent with strong reliance on a particular diet, our models suggested constrained foraging on *Xiphophora gladiata* and *Macrocystis pyrifera* (or invertebrate consumers of these algae) at ~7–13 cm ETL (Figure 5b; McCarthy 2025). This result is intriguing but derived from a limited number of observations, and warrants more extensive investigation in other *O. pullus* populations. Importantly, given the isotopic variability of local macroalgae, trophic estimates in *O. pullus* that suggested incomplete macroalgal herbivory do not unambiguously indicate feeding in the planktonic food web (Figure S5). Instead, young *O. pullus* likely shifted among different mixes of small invertebrate grazers and isotopically distinct algae (especially epiphytic rhodophytes), opportunistically blending available diet items to maximize growth (Clements and Choat 1993; Baker et al. 2016; Johnson et al. 2017). Algal assemblages that supported *O. pullus* development might be identifiable from lens chronologies using a mixing model with taxon‐specific source pools (Udy et al. 2019), a potentially fruitful approach for future applications in herbivorous fish.

For all three species, comparisons of eye lens chronologies among individuals yielded detailed insights into size‐structured patterns of variability in resource‐use ontogeny. The largest individual deviations from average model fits in small *N. fucicola* and *P. colias* were steep declines in trophic estimates (e.g., NOFU 015, PACO 046, Figure S6), which might signify movement from settlement habitats with markedly different isotopic baselines or prey assemblages. Early foraging within the Otago Harbour, a known fish nursery with elevated δ^15^N baselines relative to the open coast, could potentially explain such large positive residuals during trophic development (Currie et al. 2024; McCarthy 2025). Estimated deviations in basal resource shifts for larger *N. fucicola* were occasionally substantial but irregular, possibly reflecting inconsistencies in diet seasonality during individual growth (Denny and Schiel 2001). Among *P. colias* , individual chronologies varied in the height and within‐lens location of modeled peaks in both trophic metrics (for basal resource use, the “carbon bump”), plausibly associated with diversity in timing of recruitment into kelp forest food webs, post‐settlement migration pathways, or availability of macroalgae‐derived prey in early development (Figure 4; Figure S6). Similarly, among‐individual differences in modeled trophic peaks for *O. pullus* likely correspond to variation in the uptake rate and overall extent of juvenile omnivory, which can strongly inform physiological condition and ecological success in herbivorous fish (Clements and Choat 1993; Johnson et al. 2020). Importantly, not every lens was delaminated at the same radii or frequency, and fine‐scale patterns may have been obscured from coarser trophic chronologies (see PACO 037, Figure S6; Wallace et al. 2014). Future attempts to specify drivers of individual variability in eye lens isotopic chronologies would benefit from use of a protocol where lenses were sampled at similar intervals, in conjunction with detailed description of local isotopic baselines among habitats and diet items (Vecchio and Peebles 2020).

Aggregated patterns in lens‐derived estimates of trophic plasticity and resource‐use breadth varied by fish size and species (Figure 5). Broadly, early trophic shifts modeled for carnivorous study species were relatively consistent (low GAMM CI width, Figure 5a) despite originating from isotopically diverse post‐settlement diets (high SEA_c_, Figure 5b). Incentives to escape predation are particularly intense for small fish, which can strongly select for homogenous feeding strategies that maximize growth rates (Werner and Hall 1988). Simultaneously, morphological limitations (e.g., dentition, gape size) are likely to further constrain size‐structured dietary transitions in early development (Romanuk et al. 2011). To this point *O. pullus* , which do not feed on a strongly gape‐limited diet as juveniles (Clements and Choat 1993), exhibited the highest flexibility in modeled trophic shifts at small sizes among sampled species (Figures 4 and 5a). Conversely, estimates of growth‐related changes in *N. fucicola* trophic position were the most consistent of studied fishes, even at larger sizes (Figure 5a; Figure S6), with scope for individual variability likely minimized by their specialized dentition and selective consumption of invertebrates (Denny and Schiel 2001; Davis and Wing 2012). Estimates of resource‐use breadth for all three species reached low plateaus preceding assumed onset of maturity (~25 cm TL, Figure 5b; Morrison et al. 2014; Trip et al. 2014), possibly indicating focused foraging on fewer, higher‐quality, and larger diet items to meet the intense physiological demands of reproduction (Scharf et al. 2000; Barbini and Lucifora 2016). Opportunistic feeding on larger‐bodied prey including fish or squid is plausible for mature *P. colias* (Jiang and Carbines 2002; Beer and Wing 2013), and would be consistent with modeled shifts towards adult reliance on planktonic productivity (Figure 4; Figure S6). Similarly, the estimated length where *O. pullus* chronologies converged towards total herbivory generally aligned with the size where adults develop morphological adaptations to facilitate ingestion and digestion of nutrient‐rich phaeophytes, supporting gonadal investment (Clements and Choat 1993; Johnson et al. 2017).

Contractions in estimated resource‐use breadth around size‐at‐maturity (20 cm ETL) coincided with an apparent divergence of diets by the carnivorous species, with larger *P. colias* feeding in the same food web but at a lower average trophic position than *N. fucicola* (Figures 4 and 6). Adult trophic differentiation could indicate niche partitioning in response to interspecific competition among sympatric individuals, resulting in reduced exploitation of shared resources. However, diet composition in larger *N. fucicola* might not have been heavily influenced by present‐day interaction with *P. colias* (or other generalists), instead reflecting use of specialized dentition that evolved in response to historic competition (Connell 1980; Alley 1982; Davis and Wing 2012). In other settings, trophic relationships between *N. fucicola* and *P. colias* have been observed to differ by region, likely reflecting local macroalgal density, prey availability, and environmental drivers of foraging behavior (Udy et al. 2019). Ultimately, discerning the influence of present or past competition on our results would require detailed sampling of *P. colias* and *N. fucicola* across gradients of interaction, including surveys of resource availability, physiological condition, and relative abundance of both species as well as other putative competitors (Davis and Wing 2012; Curtis et al. 2017, 2020). Still, within the observed community, isotopic analyses of eye lenses provided useful evidence for stage‐structured changes in feeding morphology and life history incentives as regulators of interspecific trophic overlap, mediated by growth‐related shifts in individual variability of resource use.

Key technical assumptions underpin our analyses, particularly that isotopic averages of producers in our two‐source mixing model are accurate at the spatial scale of foraging by sampled fishes and temporal scale of consumer tissue turnover. Our macroalgal samples were collected across multiple seasons and coastal sites, and appear to have generated appropriate isotopic averages given the close alignment with average δ^13^C values of muscle tissue from herbivorous *O. pullus* (accounting for trophic enrichment; Figure S5). Conversely, our SPOM samples were collected during a single month and at least 25 km offshore from sampled fish populations (Figure 1). In some systems, inshore phytoplankton can yield elevated δ^13^C values relative to offshore environments, especially when conditions support large blooms and rapid cell growth (Fry and Wainright 1991; Page et al. 2008). Where similarly stratified, exclusive sampling of offshore SPOM to parameterize isotopic mixing models may systematically underestimate planktonic contributions to basal resource use (Miller and Page 2012). The Otago coastline features strong upwelling events and large phytoplankton blooms, which could drive ephemeral pulses of productivity that are ^13^C‐enriched relative to our mixing model average (Fry and Wainright 1991; Johnson et al. 2023). However, average δ^13^C values from our offshore SPOM samples were within 1‰ of phytoplankton measurements from the Otago Harbour (McCarthy 2025), and our δ^13^C offset between SPOM and macroalgae closely resembles other regional estimates including from inshore reefs (~6.5‰, Figure S5; Udy et al. 2019; Kolodzey et al. 2023). Nevertheless, related research would benefit from long‐term monitoring of local isoscapes, which provide clear frames of reference for interpretation of patterns measured in consumer tissues (e.g., Kurth et al. 2019; Faletti and Stallings 2021; Vecchio and Peebles 2022).

Although available evidence suggests that our modeling assumptions were generally valid, we still encourage caution when referring to absolute trophic estimates (% macroalgal reliance, trophic position) for a given sample, which carry substantial uncaptured uncertainty. Specifically, a 1‰ shift in producer δ^13^C averages would yield a ~15% change in proportional estimates of basal resource use, while inaccuracy of 1‰ in δ^15^N baselines would bias estimates of trophic position by ~0.3 using a δ^15^N trophic enrichment factor of 3.4‰ (Equations 1 and 2). Uncertainty in trophic enrichment factors and isotopic composition of source pools has occasionally been propagated into modeling of lens chronologies using *MixSIAR* (Young et al. 2022; Bastos et al. 2024). While this and similar tools can effectively generate group‐level summaries of trophic characteristics, current functions do not readily provide estimates for individual samples required for our analyses. Continued statistical advancements for treatment of autocorrelated isotopic data, including extrapolation of within‐source and enrichment factor uncertainty, will substantially expand capacity for ecological investigation using eye lens chronologies.

Accurate calibrations are also essential for precise trophic reconstruction from lens measurements, including the translation of within‐lens position to estimates of fish size during isotopic fixation for a given layer (Quaeck‐Davies et al. 2018). Our data corroborate descriptions of close linear relationships between lens size and fish length in larger individuals, with some non‐linearity at smaller sizes where lens deposition appears to proportionally outpace somatic growth (Figure 2; Simpson et al. 2019; Curtis et al. 2020; Vecchio et al. 2021). Reasonable extrapolations of best‐fit regression models for *P. colias* and *N. fucicola* suggest that lens growth might occasionally slow in early development for some species, skewing isometric estimates of fish size during formation of innermost laminae. Especially as eye lenses are particularly valuable in their ability to record juvenile foraging activity (Figure 4; Young et al. 2022; Bastos et al. 2024), targeted validation of relative lens diameters at small fish sizes (< 10 cm TL) remains a key research objective. As larval and recently settled specimens are often difficult to obtain, study of early lens development will likely require dedicated rearing experiments (*sensu* Granneman 2018) and microchemical cross‐referencing with other sequentially‐deposited tissues such as otoliths (Kurth et al. 2019; Stounberg et al. 2022; Diallo and Olden 2025).

Outputs of lens growth models are strongly informed by choice of calibration points (whole lens, hardened lens, or within‐PAL), with increasing differentiation at larger lens radii (Figure S4). For instance, material sampled from a *P. colias* lens at 2 mm radius corresponded to 37 cm ETL using a hardened lens calibration, but only 19 cm ETL using a whole lens approach. Conversely, estimates of fish length below 1 mm lens radius differed by less than 5 cm ETL among tested calibration models. Use of a within‐PAL calibration seems to have yielded reliable reconstructions of fish length in our study species based on the alignment of modeled trophic chronologies with known patterns of size‐structured resource use. Still, even minor uncertainty may limit the utility of eye lens analysis for applications requiring precise pairings of historic fish size with isotopic records, such as identification of spawning origins or juvenile migration pathways (Vecchio and Peebles 2020; Bell‐Tilcock et al. 2021). Continued study of metabolic activity in PAL throughout development will improve understanding of the radial threshold where isotopic records are functionally preserved, supporting further refinement of delamination protocols and lens growth models.

Our measurements also highlight the importance of calculating species‐specific adjustments for isotopic discrimination between eye lenses and reference tissues before use of trophic mixing models. For four (of six) species‐isotope pairs, we measured average PAL enrichment of ~1‰ relative to muscle, consistent with modest offsets observed across several elasmobranchs and teleosts (Table 2; Quaeck‐Davies et al. 2018; Faletti and Stallings 2021; Bastos et al. 2024). Other studies of lens isotopic composition have occasionally measured discrimination factors exceeding 4‰, particularly when comparing muscle to combinations of PAL and outer hardened lens layers (Bell‐Tilcock et al. 2021; Young et al. 2022; Diallo and Olden 2025). While not so extreme, enrichment of PAL δ^15^N relative to *O. pullus* muscle tissue was substantially higher than average offsets in our other study species (2.2‰), possibly enhanced by relative inefficiency of nitrogen assimilation and reliance on hindgut fermentation to meet energy demands (Johnson et al. 2012; Busst and Britton 2016; Baker et al. 2016). Though metabolic drivers of isotopic enrichment in PAL remain obscure, the need for their consideration in this case is clear: trophic modeling from *O. pullus* lens samples with uncorrected δ^15^N values would have identified this primary herbivore as essentially carnivorous.

Isotopic offsets between PAL and muscle tissue of *N. fucicola* were tightly and positively related to body size (Figure 3, Table 3). Rates of isotopic discrimination and tissue turnover can change as growth and metabolism slow in larger fish, though a strictly physiological mechanism would probably have driven similar associations between lens‐muscle offsets and size in *P. colias* and *O. pullus* (Suring and Wing 2009; Vander Zanden et al. 2015). Notably, feeding and growth rates in *N. fucicola* exhibit marked seasonal variation, peaking during warmer months when readily consumable prey such as amphipods and salps are abundant (Denny and Schiel 2001; Welsford and Lyle 2005). Reductions in synchronicity of lens and muscle deposition throughout growth could plausibly have enhanced isotopic offsets in large *N. fucicola* , with each tissue integrating dietary signals across different periods of variable foraging (Trueman et al. 2005; Young et al. 2022; Diallo and Olden 2025). In the end, the similarity of modeled trophic growth curves between *N. fucicola* and *P. colias* (Figure 4) suggests that adjusting lens isotopic values using a size‐invariant correction was appropriate, raising further questions regarding PAL turnover and preservation in lens archives. Through rearing studies and wild sampling, associations between fish size, isotopic composition of lens tissue, and growth or diet seasonality warrant continued investigation to better understand their causes and analytic implications.

Using stable isotope records in eye lenses from 63 individuals, we produced estimates of size‐structured resource use across complete lifetimes of three kelp forest fishes, providing particular insight into early trophic development. In summary, our data suggest that enhanced feeding on macroalgae‐derived prey could have supported rapid trophic growth and juvenile dispersal in *N. fucicola* and *P. colias* . After recruitment to coastal habitats, the onset of maturity and expansion of gape size for these carnivores appears to have allowed assumption of food web positions that were stable relative to early life, yet sufficiently plastic to accommodate modest fluctuations in resource availability. Meanwhile, our results are consistent with substantial reliance by *O. pullus* on macroalgal productivity throughout life history, despite apparent flexibility in juvenile trophic ontogeny. While relying on fewer specimens, study of eye lenses broadly corroborated muscle‐based descriptions of population‐level resource shifts at fish sizes present in coastal sampling sites. Isotopic analysis of eye lenses also yielded trophic estimates that were higher resolution and more extensive than provided by paired muscle tissue, allowing comprehensive assessment of trends, individual variability, and interspecific overlap in resource use throughout post‐settlement growth. Refined protocols for trophic modeling from lens tissue will be developed through inclusive study of species featuring diverse morphologies, diets, and life history strategies. With continued application in varied contexts, analysis of eye lens isotopic chronologies can provide fertile ground for testing hypotheses regarding trophic ontogeny at individual, population, and community scales.

## Author Contributions

**Joseph S. Curtis:** conceptualization (equal), data curation (lead), formal analysis (lead), investigation (lead), methodology (lead), visualization (lead), writing – original draft (lead), writing – review and editing (lead). **Thomas M. Chapple:** investigation (supporting), writing – review and editing (supporting). **Sophie F. Whittall:** investigation (supporting), writing – review and editing (supporting). **Leonardo M. Durante:** data curation (supporting), investigation (supporting), resources (supporting), writing – review and editing (supporting). **Gretchen J. McCarthy:** investigation (supporting), resources (supporting), writing – review and editing (supporting). **Peter W. Dillingham:** formal analysis (supporting), supervision (supporting), writing – review and editing (supporting). **Stephen R. Wing:** conceptualization (equal), formal analysis (supporting), funding acquisition (lead), methodology (supporting), supervision (lead), writing – original draft (supporting), writing – review and editing (supporting).

## Funding

This work was supported by MBIE Endeavour Project: Tau ki ākau: Ridge to Reef (UOWX2206) and National Science Challenge: Sustainable Seas.

## Conflicts of Interest

The authors declare no conflicts of interest.

## Supporting information


**Table S1:** AIC scores assessing fits of four lens growth models for each species. ΔAIC was used to select between a non‐linear and linear fit (AIC_Power_–AIC_Linear_) or between two non‐linear fits (AIC_Log‐quadratic_–AIC_Log‐linear_). Selected models and parameters are provided in Table 2.
**Table S2:** Output from linear models and GAMs assessing patterns in muscle‐derived trophic estimates (*P*_Macro_: basal resource mixture, *T*: trophic position) against fish length (cm). Outputs for GAMs describe the fit of a smooth function, with an *edf* value of 1 approximating a linear relationship and higher values indicating greater non‐linearity. ΔAIC values indicating instances where smooth functions were favored over linear models are bolded (AIC_LM_–AIC_GAM_ >> 2).
**Figure S1:** Distributions of proportional thickness of hardened cortical lens (excluding PAL) relative to whole lens diameter (including PAL), summarized using Tukey box plots.
**Figure S2:** Proportional thickness of hardened lens layers relative to lens diameter. Trendlines were generated using default LOESS regression in ggplot2 (Wickham et al. 2019). Dashed lines indicate the cutoff below which data were excluded for estimation of layer thickness (1.5 mm).
**Figure S3:** Distributions of proportional layer thickness within hardened lenses of each species. Distributions are summarized using Tukey box plots.
**Figure S4:** Comparison of competing models for estimating fish length during isotopic fixation of material at a given lens radius. Plotted curves were generated by best‐fit lens growth models for each species (Table S1) using hardened lens diameter (Inner), the estimated boundary of the layer forming within the PAL (Mid‐PAL, used in the study), and the entire lens (Whole) as calibration points. Scales vary by species.
**Figure S5:** Isotopic composition of producers (macroalgae, *n* = 109; suspended particulate organic matter or SPOM; *n* = 7) and fish tissues from three species. Mean values (±SD) are plotted over raw data. Lines extending from producers indicate expected isotopic increases with trophic position, with slopes determined by estimated enrichment factors per trophic level (δ^13^C: 0.4‰, δ^15^N: 3.4‰). Genera of sampled macroalgae included *Cystophora*, *Dictyota*, *Durvillaea*, *Lessonia*, *Macrocystis*, *Marginariella*, *Ulva*, *Undaria* and *Xiphophora*. Excluded macroalgae included genera known to contain highly unpalatable compounds (*Caulerpa*, *Desmarestia*), as well as nine samples that were identified as within‐genus outliers using Tukey criteria. One SPOM outlier was also excluded.
**Figure S6:** Individual lens‐based chronologies of trophic estimates (top: basal resource mixture or proportional contribution of macroalgae; bottom: trophic position) plotted against GAMM predictions (smooth lines), which incorporate the global smooth fit (per species) and a random intercept for individual fish. Deviations from predicted fits can be interpreted as an alternate ontogeny of resource use to the ‘average’ fish.

## Data Availability

Data and novel code supporting our analyses are archived at: https://doi.org/10.6084/m9.figshare.29431916.
