# Supplementary material for: Stable Isotopes in Eye Lenses Record Patterns and Variation in Resource‐Use Ontogeny of Three New Zealand Kelp Forest Fishes
Source: Ecol Evol. 2026 Jul 9;16(7):e73921. doi: 10.1002/ece3.73921 (PMC13349662; doi:10.1002/ece3.73921)
Supplement: Supplementary file 1 — Table S1: AIC scores assessing fits of four lens growth models for each species. ΔAIC was used to select between a non‐linear and linear fit (AICPower–AICLinear) or between two non‐linear fits (AICLog‐quadratic–AICLog‐linear). Selected models and parameters are provided in Table 2. Table S2: Output from linear models and GAMs assessing patterns in muscle‐derived trophic estimates (P Macro: basal resource mixture, T: trophic position) against fish length (cm). Outputs for GAMs describe the fit of a smooth function, with an edf value of 1 approximating a linear relationship and higher values indicating greater non‐linearity. ΔAIC values indicating instances where smooth functions were favored over linear models are bolded (AICLM–AICGAM >> 2). Figure S1: Distributions of proportional thickness of hardened cortical lens (excluding PAL) relative to whole lens diameter (including PAL), summarized using Tukey box plots. Figure S2: Proportional thickness of hardened lens layers relative to lens diameter. Trendlines were generated using default LOESS regression in ggplot2 (Wickham et al. 2019). Dashed lines indicate the cutoff below which data were excluded for estimation of layer thickness (1.5 mm). Figure S3: Distributions of proportional layer thickness within hardened lenses of each species. Distributions are summarized using Tukey box plots. Figure S4: Comparison of competing models for estimating fish length during isotopic fixation of material at a given lens radius. Plotted curves were generated by best‐fit lens growth models for each species (Table S1) using hardened lens diameter (Inner), the estimated boundary of the layer forming within the PAL (Mid‐PAL, used in the study), and the entire lens (Whole) as calibration points. Scales vary by species. Figure S5: Isotopic composition of producers (macroalgae, n = 109; suspended particulate organic matter or SPOM; n = 7) and fish tissues from three species. Mean values (±SD) are plotted over raw data. Lines extending f [file ECE3-16-e73921-s001.docx]

**Supporting Information**: **Stable isotopes in eye lenses record patterns and variation in resource-use ontogeny of three New Zealand kelp forest fishes**

Joseph S. Curtis^1^*, Gretchen J. McCarthy^1^, Leonardo M. Durante^1^, Thomas M. Chapple^1^, Sophie F. Whittall^1^, Peter W. Dillingham^2^ and Stephen R. Wing^1^

^1^Department of Marine Science, University of Otago, Dunedin, Otago, New Zealand, 9054

^2^Department of Mathematics and Statistics, University of Otago, Dunedin, Otago, New Zealand, 9054

*Corresponding author: [joseph.curtis@otago.ac.nz](mailto:joseph.curtis@otago.ac.nz)

*Lens size calibration*

We estimated historic fish length from measurements of lens radius using a calibration point (C) that approximated the boundary of the layer forming within outer lens material, or pre-apoptotic laminae (PAL; Kuntz et al. 2025). The boundary between PAL and inner hardened lens material was identified based on notes regarding lens texture and the radius at which cortical material was unambiguously dehydrated (Fig. S1)*.* In all three species, the PAL-hardened lens boundary (transition zone) was estimated at a consistent relative position (mean ±SD; *Notolabrus fucicola*: 67.7 ±6.6% lens radius; *Odax pullus*: 66.7 ±3.7%; *Parapercis colias*: 67.7 ±4.4%; Fig. S1)*.* This matches closely with previous estimates of proportional PAL thickness observed in several fish taxa (Leifsdottir and Campana 2023, Kuntz et al. 2025).

To estimate C, the approximate radial position of the transition zone for each lens (the product of per-species average PAL proportional thickness and whole lens diameter, Eq. 4) was added to the average proportional thickness of layers within the hardened lens (Eq. 5). We first plotted proportional thickness of hardened-lens layers against lens radius to examine whether layer thickness changed with distance from the lens center, or whether an average estimate of layer thickness was suitable for use in calibration formulae. The lens layer immediately interior of the PAL transition was excluded from this analysis, as its thickness was influenced by PAL removal success. We identified a consistent increase in proportional layer thickness below a lens diameter of ~1.5 mm, likely due to limitations of dexterity while manually delaminating very small lenses (Fig. S2)*.* We therefore calculated average proportional layer thickness using samples from lenses >1.5 mm diameter, excluding a few extreme values (layer thickness >25% lens radius) that probably resulted from excessive hydration during delamination (Fig. S3)*.* Because there was only slight species-specific variation in average proportional layer thickness, we used one value (12%) for calibration in all three species (*N. fucicola*: 10.8 ±4.3% lens radius; *O. pullus*: 12.4 ±4.4%; *P. colias*: 12.0 ±4.5%).

Using C, we estimated fish size (estimated total length, ETL) from lens radius across the observed measurement range using linear models, power models, or log-quadratic models. Models were formulated as:

Linear: $\mathrm{ETL}= \beta_{0}+\beta_{1}C$ (S1)

Power (raw-scale formulation): $\mathrm{ETL}= \beta_{0}C^{\beta_{1}}$ (S2)

Power (log-linear formulation): $\log\left( \mathrm{ETL} \right)= log(\beta_{0})+\beta_{1}\log\left( C \right)$ (S3)

Log-Quadratic: $\log\left( \mathrm{ETL} \right)= \beta_{0}+\beta_{1}\log\left( C \right)+\beta_{2}\log(C)^{2}$ (S4)

Model fits were compared using AIC scores, and the model yielding the lower AIC score was determined to provide strong indication of an improved fit when ΔAIC >>2 (Bozdogan 1987). Because AIC scores are not congruent between natural and logarithmic scales, we compared fits of linear to power models on the natural scale and power to log-quadratic models on the logarithmic scale (Table S1). Where ΔAIC did not identify a clearly favored model, we defaulted to the parsimonious option of linear lens growth.

*Supplementary Tables and Figures*

Table S1. AIC scores assessing fits of four lens growth models for each species. ΔAIC was used to select between a non-linear and linear fit (AIC_Power_ – AIC_Linear_) or between two non-linear fits (AIC_Log-quadratic_ - AIC_Log-linear_). Selected models and parameters are provided in Table 2

| ***Species*** | ***Regression*** | ***df*** | ***AIC*** | ***ΔAIC*** |
| --- | --- | --- | --- | --- |
| ***Notolabrus fucicola*** | Linear | 3 | 90.59 | -1.22 |
|  | Power | 3 | 89.37 |  |
|  | Log-linear | 3 | -52.24 | **-9.62** |
|  | **Log-quadratic** | 4 | -61.86 |  |
| ***Odax pullus*** | **Linear** | 3 | 92.35 | -0.09 |
|  | Power | 3 | 92.26 |  |
|  | Log-linear | 3 | -30.94 | 0.97 |
|  | Log-quadratic | 4 | -29.97 |  |
| ***Parapercis colias*** | Linear | 3 | 355.65 | **-9.07** |
|  | Power | 3 | 346.58 |  |
|  | Log-linear | 3 | -108.99 | **-23.29** |
|  | **Log-quadratic** | 4 | -132.28 |  |

Table S2. Output from linear models and GAMs assessing patterns in muscle-derived trophic estimates (*P*_Macro_: basal resource mixture, *T*: trophic position) against fish length (cm). Outputs for GAMs describe the fit of a smooth function, with an *edf* value of 1 approximating a linear relationship and higher values indicating greater non-linearity. ΔAIC values indicating instances where smooth functions were favored over linear models are bolded (AIC_LM_ – AIC_GAM_  >>2)

|  | | **Linear model** | | | | **GAM** | | |
| --- | --- | --- | --- | --- | --- | --- | --- | --- |
| ***Species*** | ***Response*** | ***β*** | ***SE*** | ***t*** | ***r^2^*** | ***edf*** | ***Adj. R^2^*** | ***ΔAIC*** |
| ***Notolabrus fucicola*** | *P*_Macro_ | -0.005 | 0.003 | -1.50 | 0.089 | 2.22 | 0.172 | 0.989 |
|  | *T* | -0.004 | 0.002 | -1.86 | 0.131 | 7.67 | 0.517 | **10.9** |
| ***Odax pullus*** | *P*_Macro_ | 0.004 | 0.002 | 2.16 | 0.135 | 1.00 | 0.176 | **134.6** |
|  | *T* | -0.004 | 0.002 | -1.77 | 0.098 | 2.21 | 0.157 | 2.054 |
| ***Parapercis colias*** | *P*_Macro_ | -0.003 | 0.002 | -2.00 | 0.100 | 1.49 | 0.091 | -1.685 |
|  | *T* | -0.001 | 0.003 | -0.30 | 0.002 | 1.72 | 0.024 | 1.183 |


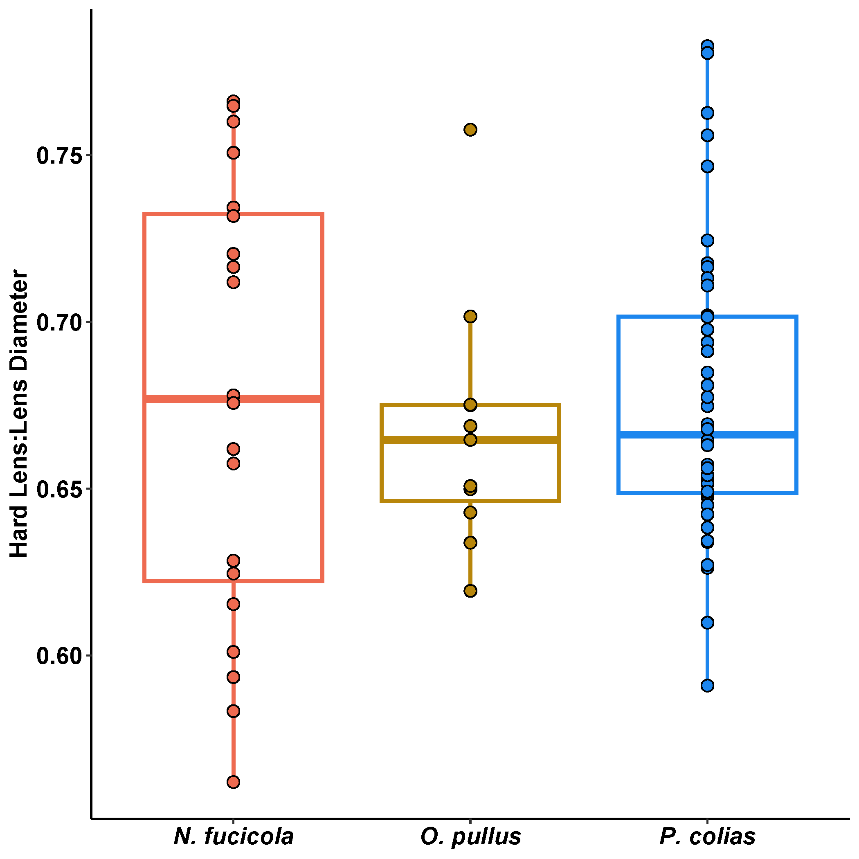


Figure S1. Distributions of proportional thickness of hardened cortical lens (excluding PAL) relative to whole lens diameter (including PAL), summarized using Tukey box plots


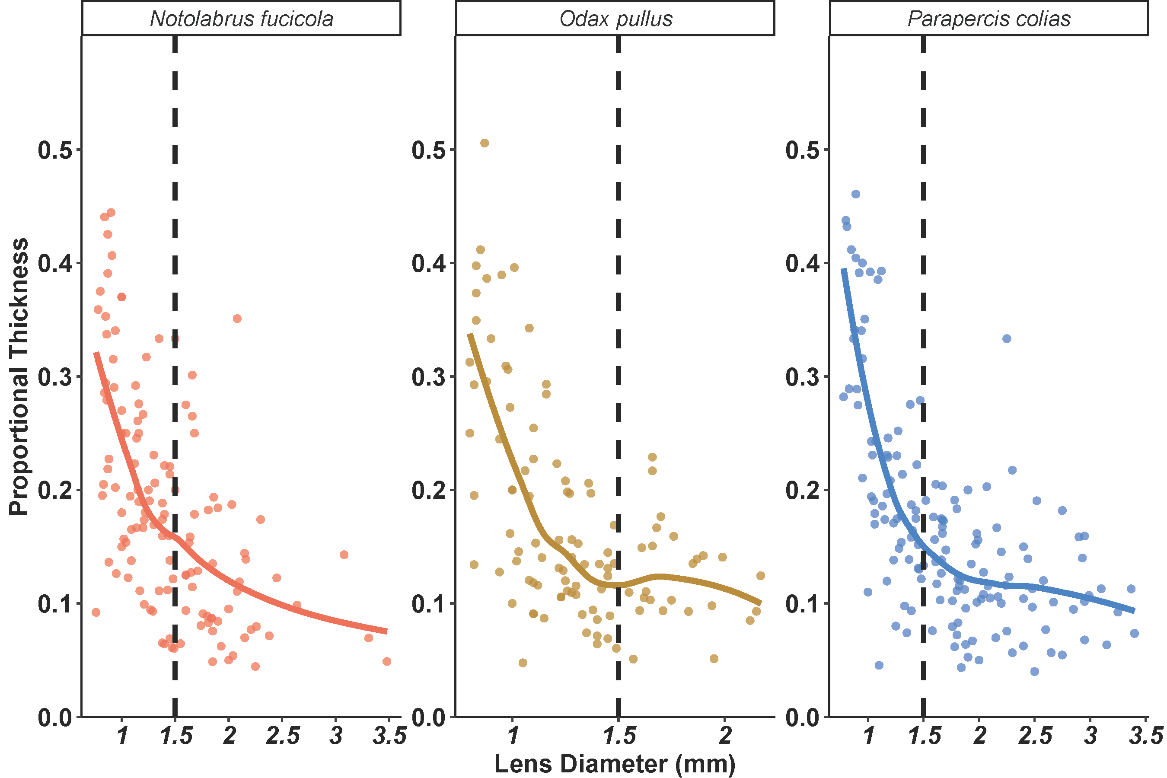


Figure S2.Proportional thickness of hardened lens layers relative to lens diameter. Trendlines were generated using default LOESS regression in ggplot2 (Wickham et al. 2019). Dashed lines indicate the cutoff below which data were excluded for estimation of layer thickness (1.5 mm)

*
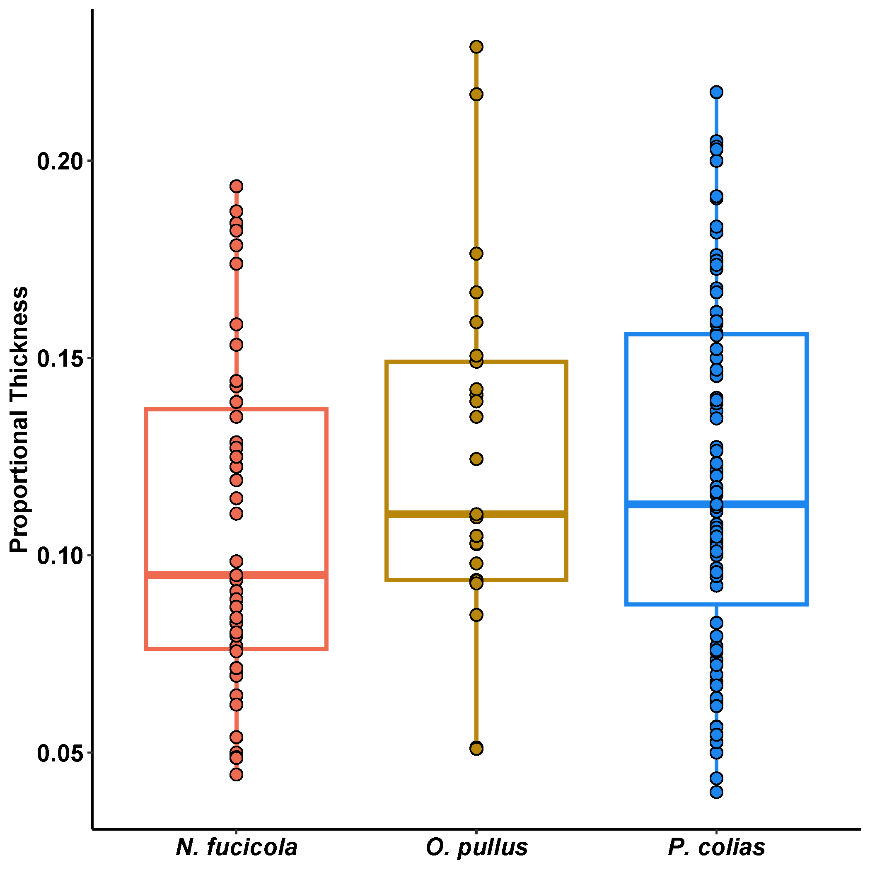
*

Figure S3. Distributions of proportional layer thickness within hardened lenses of each species. Distributions are summarized using Tukey box plots


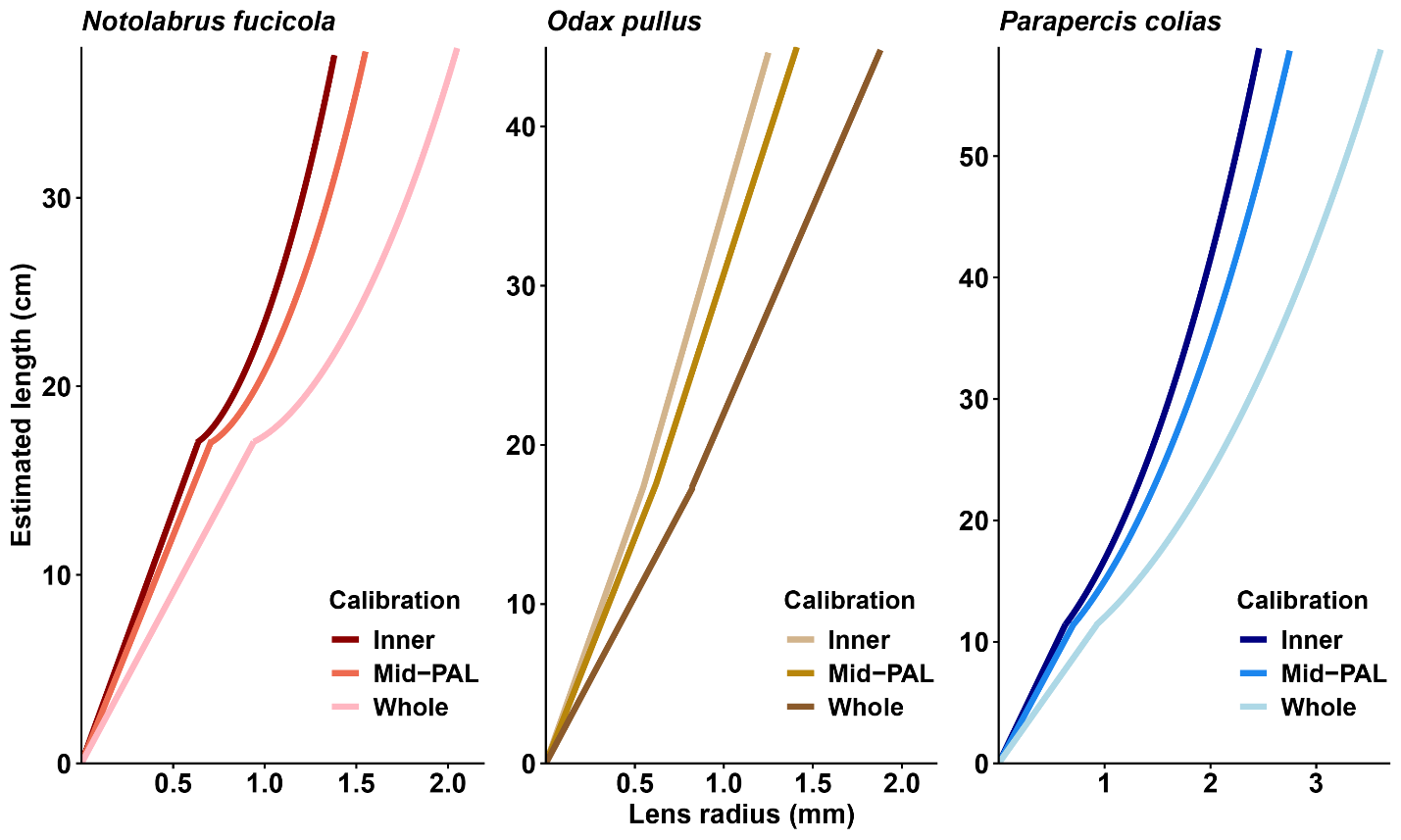


Figure S4. Comparison of competing models for estimating fish length during isotopic fixation of material at a given lens radius. Plotted curves were generated by best-fit lens growth models for each species (Table S1) using hardened lens diameter (Inner), the estimated boundary of the layer forming within the PAL (Mid-PAL, used in the study), and the entire lens (Whole) as calibration points. Scales vary by species


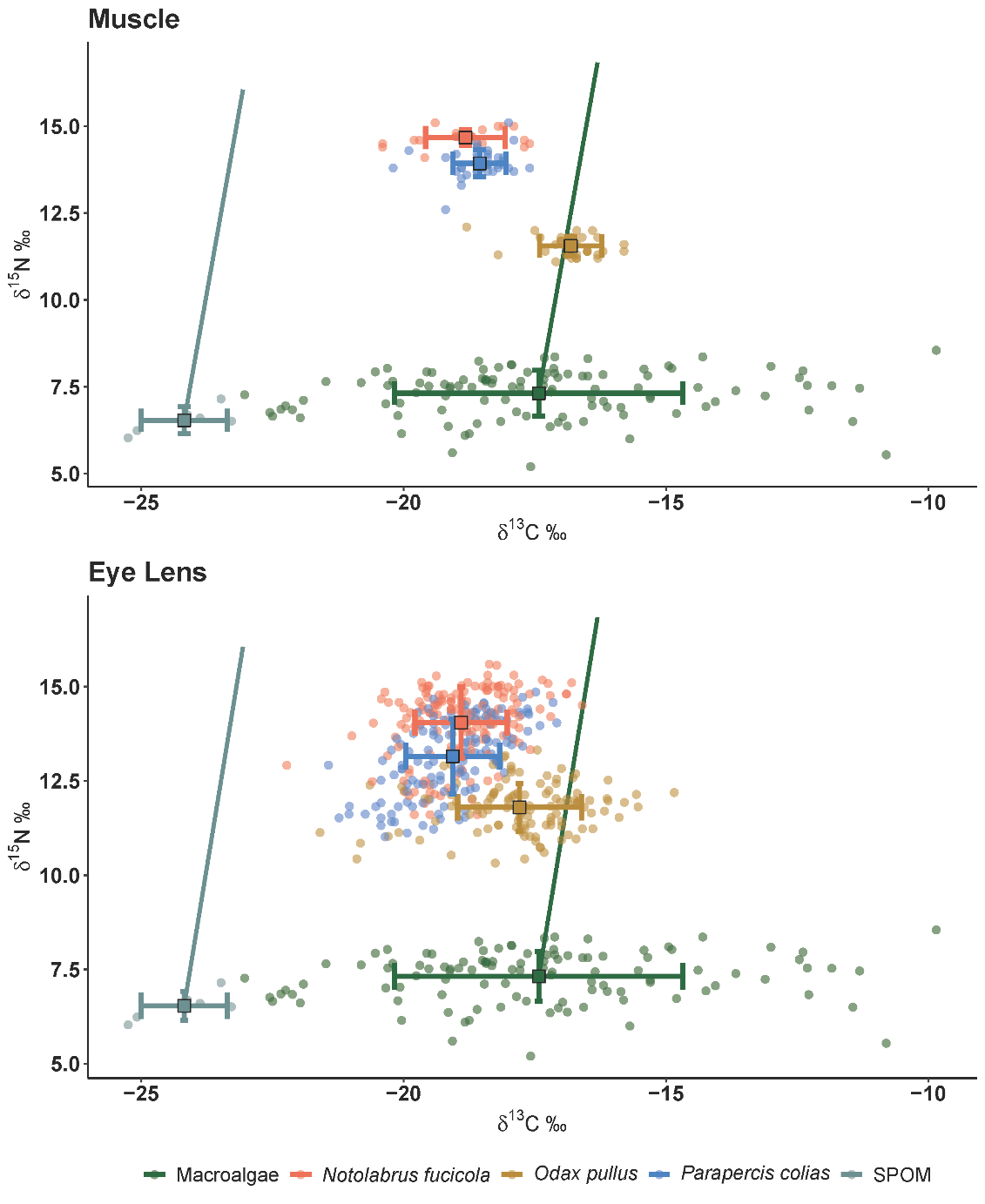


Figure S5. Isotopic composition of producers (macroalgae, n = 109; suspended particulate organic matter or SPOM; n = 7) and fish tissues from three species. Mean values (±SD) are plotted over raw data. Lines extending from producers indicate expected isotopic increases with trophic position, with slopes determined by estimated enrichment factors per trophic level (*δ*^13^C: 0.4‰, *δ*^15^N: 3.4‰)*.* Genera of sampled macroalgae included *Cystophora*, *Dictyota*, *Durvillaea*, *Lessonia*, *Macrocystis*, *Marginariella*, *Ulva*, *Undaria*, and *Xiphophora.* Excluded macroalgae included genera known to contain highly unpalatable compounds (*Caulerpa*, *Desmarestia*)*,* as well as nine samples that were identified as within-genus outliers using Tukey criteria. One SPOM outlier was also excluded


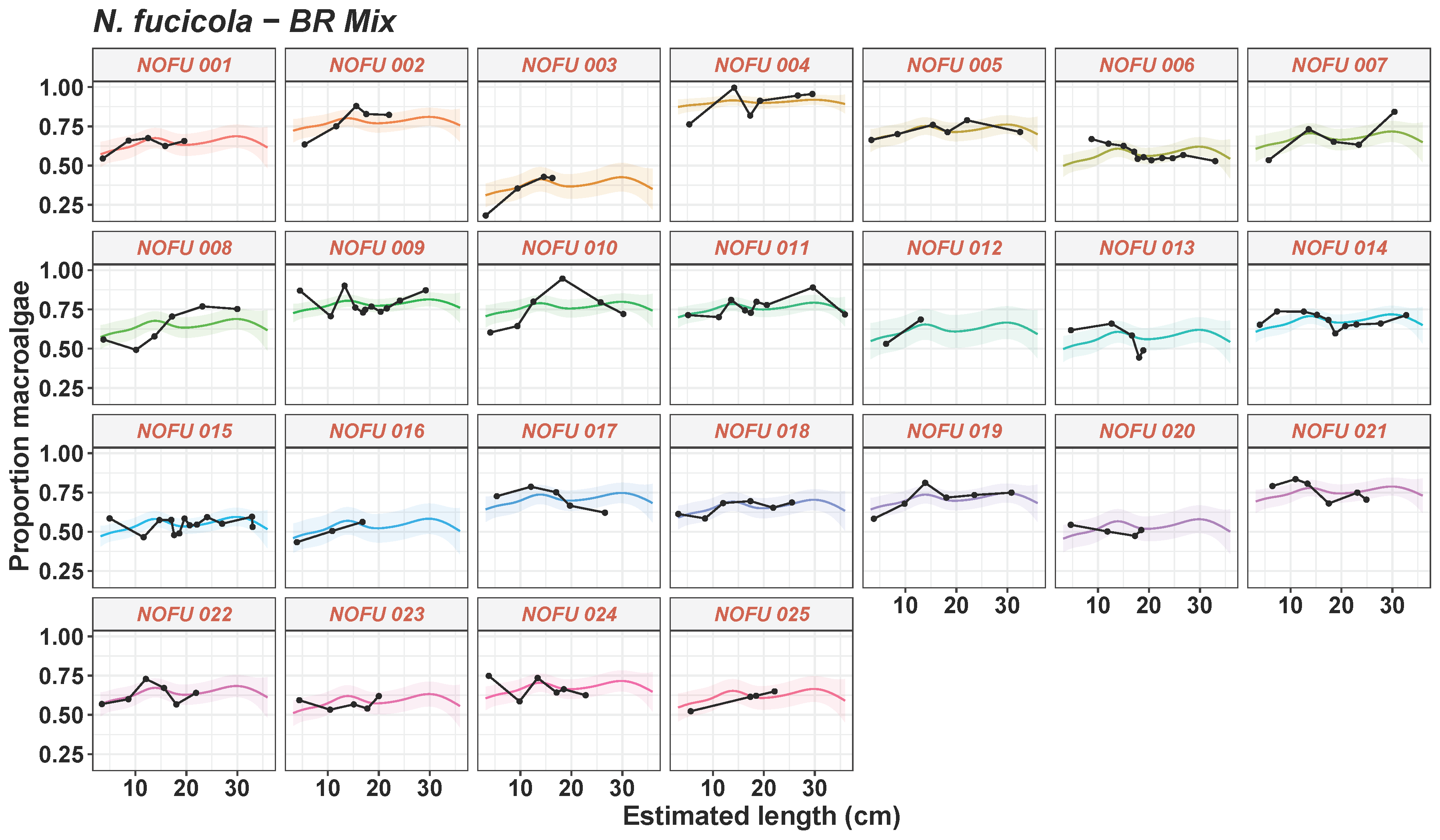


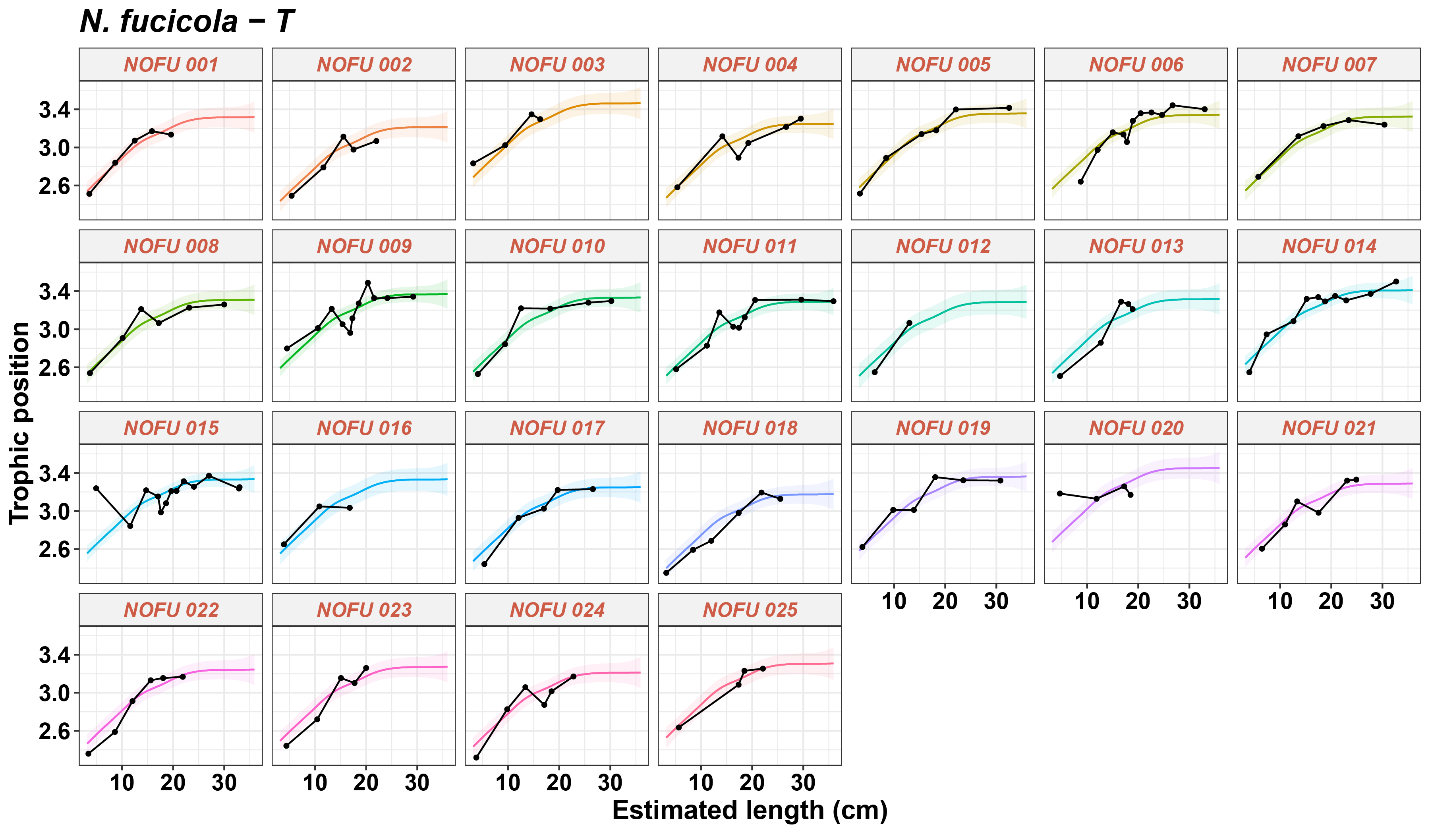
 Figure S6. Individual lens-based chronologies of trophic estimates (top: basal resource mixture or proportional contribution of macroalgae; bottom: trophic position) plotted against GAMM predictions (smooth lines), which incorporate the global smooth fit (per species) and a random intercept for individual fish. Deviations from predicted fits can be interpreted as an alternate ontogeny of resource use to the “average” fish


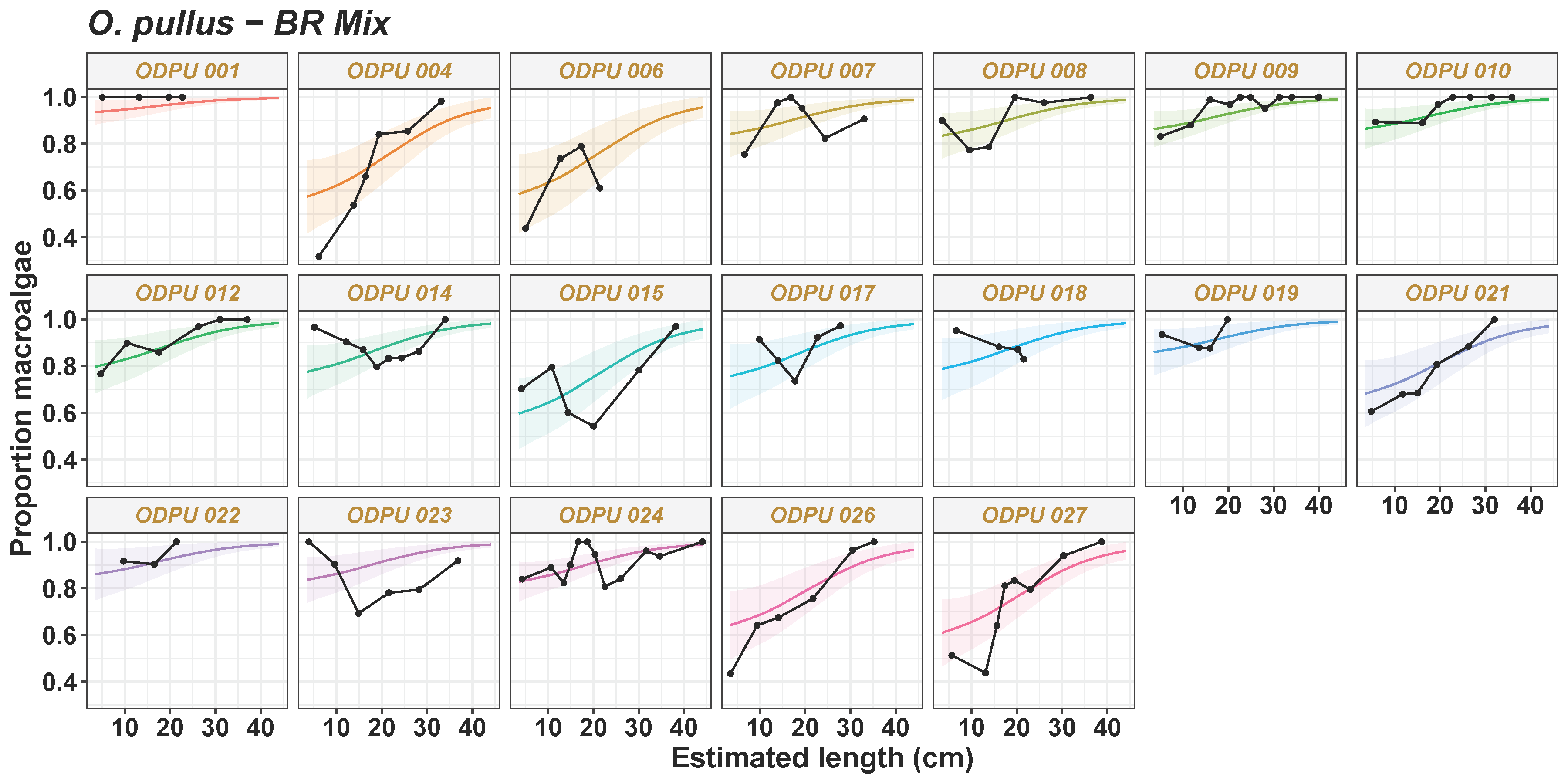

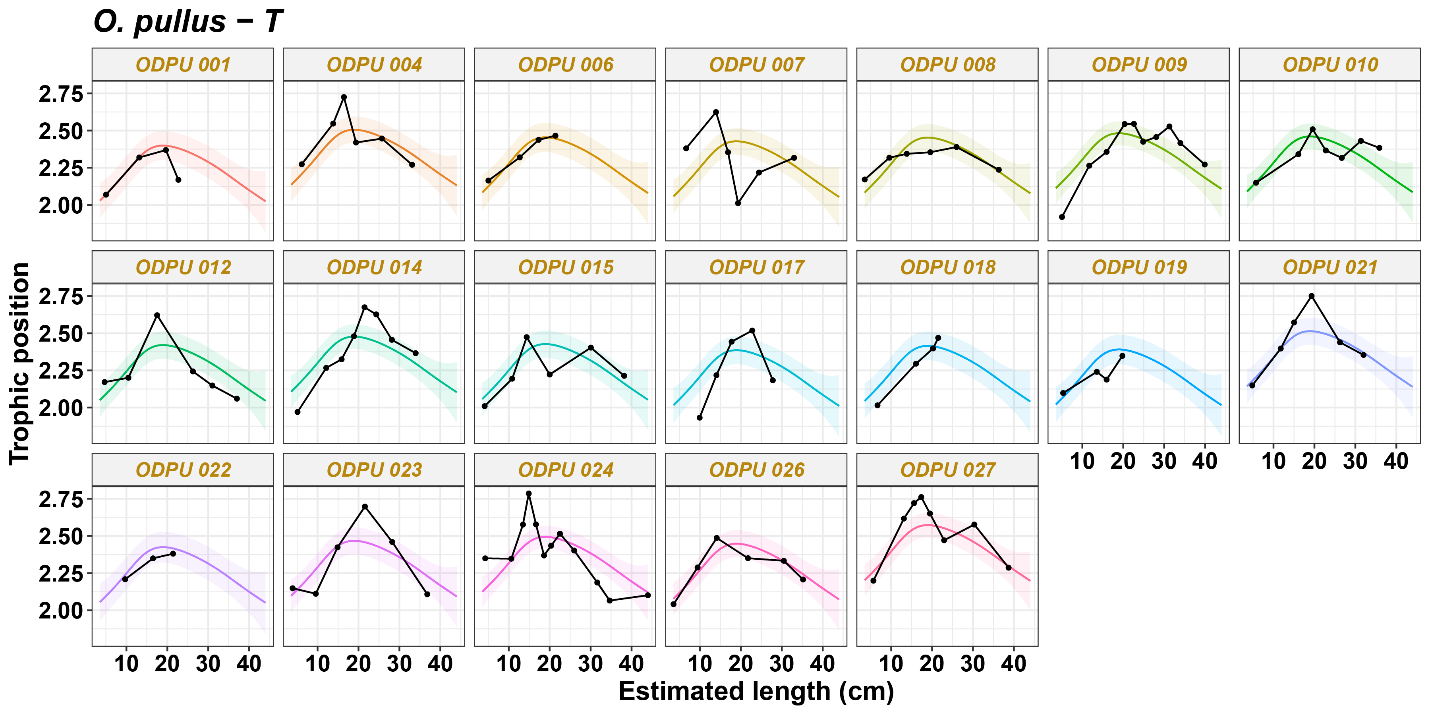


Figure S6 (cont). Individual lens-based chronologies of trophic estimates (top: basal resource mixture or proportional contribution of macroalgae: top; bottom: trophic position) plotted against GAMM predictions (smooth lines), which incorporate the global smooth fit (per species) and a random intercept for individual fish. Deviations from predicted fits can be interpreted as an alternate ontogeny of resource use to the “average” fish
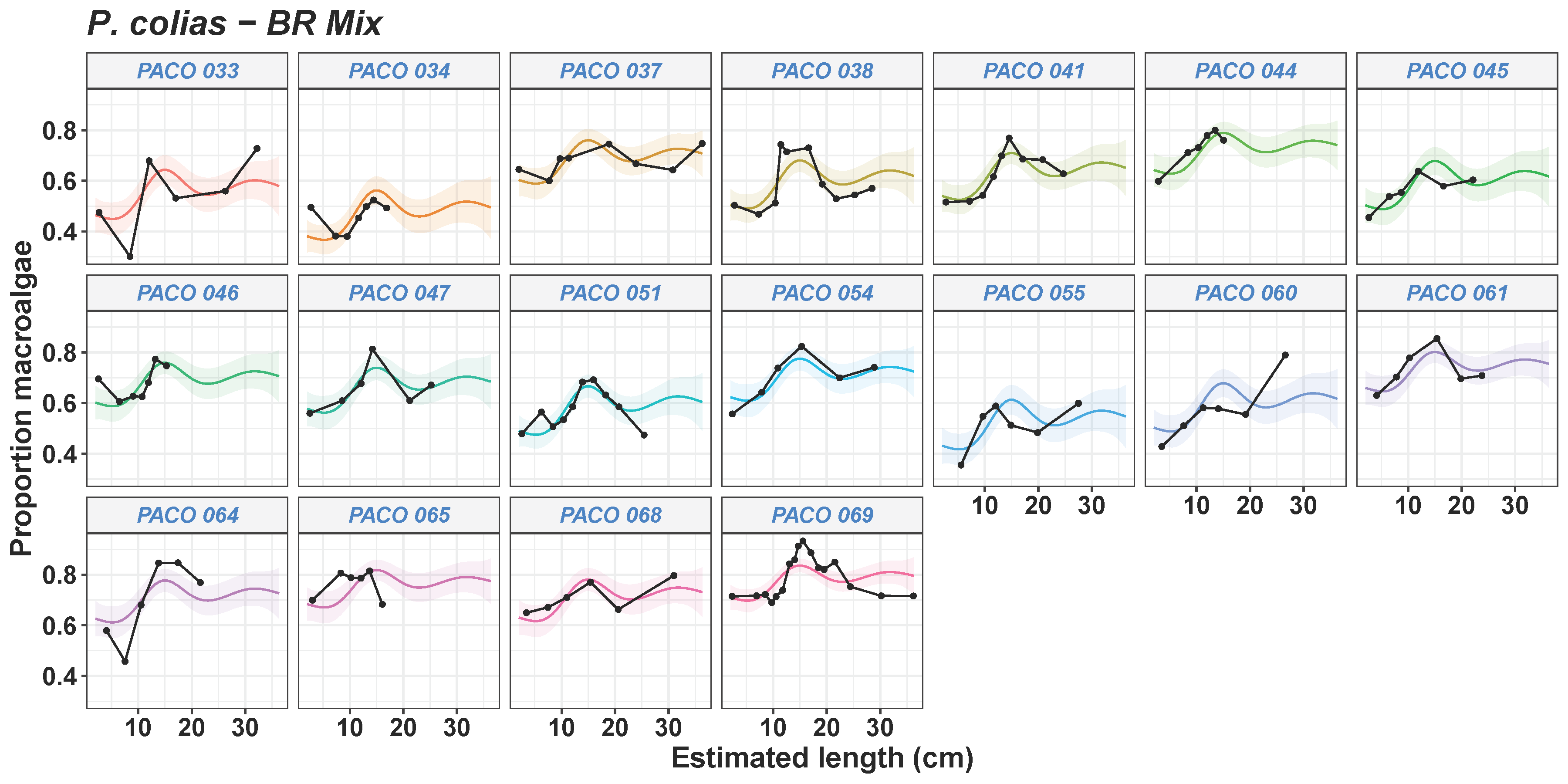

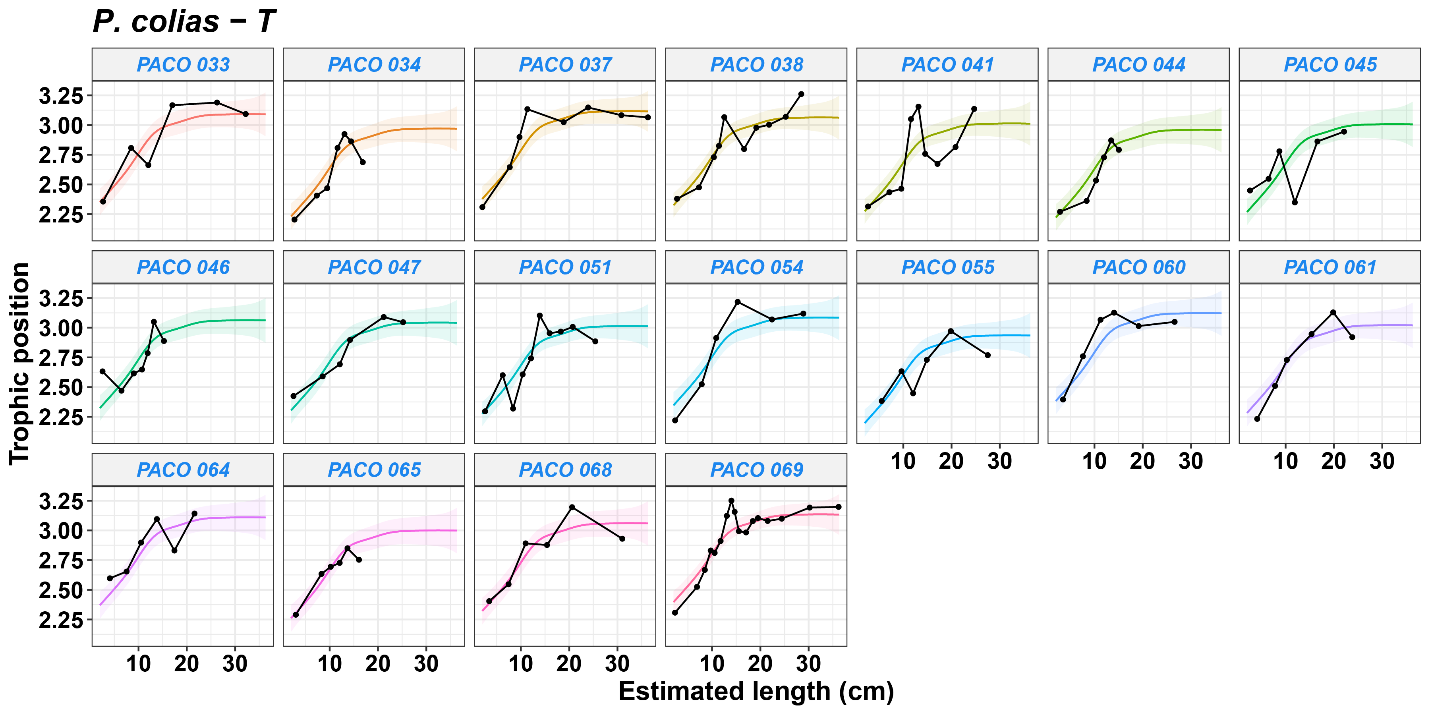


Figure S6 (cont). Individual lens-based chronologies of trophic estimates (top: basal resource mixture or proportional contribution of macroalgae: top; bottom: trophic position) plotted against GAMM predictions (smooth lines), which incorporate the global smooth fit (per species) and a random intercept for individual fish. Deviations from predicted fits can be interpreted as an alternate ontogeny of resource use to the “average” fish
